# MQM1, a bacteriophage infecting strains of *Aeromonas salmonicida* subspecies *salmonicida* carrying Prophage 3

**DOI:** 10.1016/j.virusres.2023.199165

**Published:** 2023-06-30

**Authors:** Nava Hosseini, Valérie E. Paquet, Pierre-Étienne Marcoux, Charles-Antoine Alain, Maude F. Paquet, Sylvain Moineau, Steve J. Charette

**Affiliations:** a Institut de Biologie Intégrative et des Systèmes (IBIS), Pavillon Charles-Eugène-Marchand, Université Laval, Quebec City, QC, G1V 0A6, Canada.; b Département de biochimie, de microbiologie et de bio-informatique, Faculté des sciences et de génie, Université Laval, Quebec City, QC, G1V 0A6, Canada.; c Centre de Recherche de l'Institut Universitaire de Cardiologie et de Pneumologie de Québec (IUCPQ), Quebec City, QC, G1V 4G5, Canada.; d Groupe de Recherche en Écologie Buccale (GREB), Faculté de médecine dentaire, Université Laval, Quebec City, QC, G1V 0A6, Canada.; e Félix d'Hérelle Reference Center for Bacterial Viruses, Université Laval, Quebec City, QC, G1V 0A6, Canada.

**Keywords:** Aeromonas salmonicida subsp. salmonicida, furunculosis, phage therapy, phage cocktail, Prophage 3, vB_AsaP_MQM1

## Abstract

*Aeromonas salmonicida* subsp. *salmonicida* is a Gam-negative bacterium responsible for furunculosis in fish. Because this aquatic bacterial pathogen has a rich reservoir of antibiotic-resistant genes, it is essential to investigate antibacterial alternatives, including the use of phages. Yet, we have previously demonstrated the inefficiency of a phage cocktail designed against *A. salmonicida* subsp. *salmonicida* strains due to a phage resistance phenotype associated to a prophage, namely Prophage 3. To bypass this resistance, one of the solutions is to isolate novel phages capable of infecting Prophage 3-bearing strains. Here we report on the isolation and characterization of the new virulent phage vB_AsaP_MQM1 (or MQM1), which is highly specific to *A. salmonicida* subsp. *salmonicida* strains. Phage MQM1 inhibited the growth of 01-B516, a strain carrying Prophage 3, including when combined to the previous phage cocktail. MQM1 infected 26 out of the 30 (87%) Prophage 3-bearing strains tested. Its linear dsDNA genome contains 63,343 bp, with a GC content of 50.2%. MQM1 genome can encode 88 proteins and 8 tRNAs, while no integrase or transposase-encoding genes were found. This podophage has an icosahedral capsid and a non-contractile short tail. We suggest that MQM1 may be a good addition to future phage cocktails against furunculosis to resolve the Prophage 3-resistance issue.

## Highlights

• vB_AsaP_MQM1 (or MQM1) is a newly isolated virulent podophage.
• MQM1 is highly specific to *A. salmonicida* subsp. *salmonicida* strains.
• MQM1 is capable of infecting a broad range of Prophage 3-bearing strains.
• MQM1 has no integrase or transposase-encoding genes.
• Addition of MQM1 in a phage cocktail resolves the Prophage 3-resistance issue.

## 1 Introduction

Since the beginning of the 21st century, a search of the keyword “bacteriophage” in the scientific literature shows a resurgence in the interest of phages as potential therapeutics, particularly in the context of the post-antibiotic era. Phage therapy offers several advantages, including a greater specificity towards bacterial strains causing the infection (Kortright et al., 2019). This approach is also relevant for the One Health principle, which tries to consider and accommodate the strong link between human and animal health as well as the environment (Pires et al., 2020). These reasons explain the increased interest in phage applications to address fish infections like furunculosis (Park et al., 2020).

Furunculosis is a deadly bacterial infection that specifically affects salmonid species and causes heavy annual economic losses in aquaculture worldwide (Dallaire-Dufresne et al., 2014). The causal agent of furunculosis is a psychrophilic bacterium named *Aeromonas salmonicida* subsp. *salmonicida.* In addition, several strains belonging to this bacterial species are resistant to most antibiotics and in some cases to all approved antibiotics for aquaculture in Canada (Trudel et al., 2016). The antibiotic resistance genes are most often carried on a large set of diverse plasmids (Vincent et al., 2021). In addition to plasmids, other mobile genetic elements (MGEs) occur frequently in *A. salmonicida* subsp. *salmonicida*. It is particularly the case for prophage-derived genomic islands (*AsaGEI*s), and prophages (Emond-Rheault et al., 2015a; Vincent et al., 2021b). Interestingly, these MGEs appear to be geographically specific, as is the case for *AsaGEI1a* and *AsaGEI2a* which are found in strains isolated in North America (Emond-Rheault et al., 2015a). Unlike Prophage 1 and Prophage 2 which have been found in all *A. salmonicida* subsp. *salmonicida* strains, Prophage 3 is limited to many North American strains. For example, Prophage 3 has been detected by PCR genotyping in about ⅔ of the *A. salmonicida* subsp. *salmonicida* strains isolated from the Province of Quebec (Canada), and it is always located in the same genomic region, between *AsaGEI2a* and a tRNA_Leu_ gene. A small fraction of strains has *AsaGEI2a* but not Prophage 3 (Emond-Rheault et al., 2015a)*.*

Previously, we designed a phage cocktail (i.e. a combination of phages with a complementary lytic spectrum) comprising four lytic myophages, three isolated in Canada (two in Quebec and one in Ontario) and one from France (Saône-et-Loire) (Vincent et al., 2017). This phage cocktail proved to be effective *in vitro* to inhibit the growth of various *A. salmonicida* subsp. *salmonicida* strains when used at an initial multiplicity of infection (MOI) of 1. However, the same phage cocktail was ineffective for inhibiting the growth of *A. salmonicida* subsp. *salmonicida* strains harboring Prophage 3 (Hosseini et al., 2021). Leduc *et al.* isolated phage vB_AsaM_LPM4 from an infected fish and its complete genomic sequence is identical to Prophage 3 (Leduc et al., 2023). This temperate phage was then introduced into phage-sensitive *A. salmonicida* subsp. *salmonicida* strains and the lysogenized strain became resistant to the phage cocktail, thereby confirming the ineffectiveness of the phage cocktail against strains that bear Prophage 3 (Hosseini et al., 2021).

Here, we attempted to isolate at least one new virulent phage from environmental samples using a bacterial host that carries Prophage 3. We successfully isolated phage vB_AsaP_MQM1 (or simply MQM1) from the mucus of a dead fish obtained from a fish farm in Quebec. According to genome sequence annotation and morphological analysis, MQM1 is a virulent podophage, a morphology that has have been rarely observed for phages infecting *A. salmonicida* subsp. s*almonicida* strains (Park et al., 2020). The host range of phage MQM1 confirmed its effectiveness against most strains carrying Prophage 3. The unique host specificity of phage MQM1 makes it a promising candidate to add to a phage cocktail against furunculosis (Hosseini et al., 2021).

## 2 Materials and methods

### 2.1 Bacterial host strains

*A. salmonicida* subsp. *salmonicida* strains used in this study are listed in Table 1. Two psychrophilic *A. salmonicida* subsp*. salmonicida* strains that carry *AsaGEI2a* and Prophage 3 (01-B516 and 2004-05 MF26) were chosen as potential phage hosts. These two well-characterized strains present opposite characteristics regarding antibiotic resistance; 01-B526 has no resistance genes for antibiotics used in aquaculture while it is the case for 2004-05 MF26 (Daher et al., 2011; Marcoux et al., 2021; Vincent et al., 2015, 2014). The strains were thawed directly from 15% glycerol stocks frozen at -70°C and streaked on tryptic soy agar plates (TSA, Wisent, St-Bruno, QC, Canada). After 3 days of incubation at 18°C, 2 to 5 colonies of each strain were inoculated in 10 mL of tryptic soy broth (TSB, Wisent, St-Bruno, QC, Canada) and incubated overnight at 18°C with agitation at 200 rpm.

**Table 1:** Bacterial strains used in this study and phage MQM1 host range against A. salmonicida subsp. salmonicida.

Strain name	Origin	Reference	Sensitivity to MQM1	Prophage 3	*AsaGEI*	Gene cluster^a^	TTSS^b^	A-layer	Antibiotic resistance genes^c^
01-B516	Quebec	(Daher et al., 2011)	Yes	Yes	*2a*	No	Yes	Yes	No
57043	Quebec	(Emond-Rheault et al., 2015b)	Yes	Yes	*2a*	No	Yes	No	No
m17524-09	Quebec	(Emond-Rheault et al., 2015b)	Yes	Yes	*2a*	No	No	Yes	No
m19438-09	Quebec	(Emond-Rheault et al., 2015b)	Yes	Yes	*2a*	No	Yes	Yes	No
m11743-09	Quebec	(Emond-Rheault et al., 2015b)	Yes	Yes	*2a*	No	Yes	Yes	No
m23067-09	Quebec	(Emond-Rheault et al., 2015b)	Yes	Yes	*2a*	No	Yes	Yes	No
m23281-09	Quebec	(Emond-Rheault et al., 2015b)	Yes	Yes	*2a*	No	Yes	Yes	No
m8029-10	Quebec	(Emond-Rheault et al., 2015b)	Yes	Yes	*2a*	No	Yes	Yes	No
m19878-11	Quebec	(Emond-Rheault et al., 2015b)	Yes	Yes	*2a*	No	Yes	Yes	No
m13460-11	Quebec	(Emond-Rheault et al., 2015b)	Yes	Yes	*2a*	No	Yes	Yes	No
m13182-11	Quebec	(Emond-Rheault et al., 2015b)	Yes	Yes	*2a*	No	Yes	Yes	No
m16474-11	Quebec	(Emond-Rheault et al., 2015b)	Yes	Yes	*2a*	No	Yes	Yes	*sul*1, *flo*R, *tet*A, *tet*G
m15878-11	Quebec	(Emond-Rheault et al., 2015b)	Yes	Yes	*2a*	No	Yes	Yes	No
m22710-11	Quebec	(Emond-Rheault et al., 2015b)	Yes	Yes	*2a*	No	Yes	Yes	No
m13050-12	Quebec	(Emond-Rheault et al., 2015b)	Yes	Yes	*2a*	No	Yes	Yes	*sul*1, *sul*2, *flo*R, *tet*A
m14404-12	Quebec	(Emond-Rheault et al., 2015b)	Yes	Yes	*2a*	No	Yes	Yes	No
m12976-12	Quebec	(Emond-Rheault et al., 2015b)	Yes	Yes	*2a*	No	Yes	Yes	No
m12418-12	Quebec	(Emond-Rheault et al., 2015b)	Yes	Yes	*2a*	No	Yes	Yes	No
m21368-12	Quebec	(Emond-Rheault et al., 2015b)	Yes	Yes	*2a*	No	Yes	Yes	No
m24783-12	Quebec	(Emond-Rheault et al., 2015b)	Yes	Yes	*2a*	No	Yes	Yes	No
SHY13-162	Quebec	(Trudel et al., 2016)	Yes	Yes	*2a*	No	Yes	Yes	No
SHY13-2257	Quebec	(Trudel et al., 2016)	Yes	Yes	*2a*	No	Yes	Yes	No
2004-05 MF26	New Brunswick	(Vincent et al., 2014)	Yes	Yes	*2a*	No	Yes	Yes	*sul*1, *sul*2, *flo*R, *tet*A
2009-144 K3	New Brunswick	(Vincent et al., 2014)	Yes	Yes	*2a*	No	Yes	Yes	*sul*2, *flo*R, *tet*A(C),*tet*H
SHY15-2939	Quebec	This study	Yes	Yes	*No*	No	Yes	Yes	*sul*1
SHY18-4069	Quebec	This study	Yes	Yes	*No*	No	Yes	Yes	No
HER1107	Quebec	(Daher et al., 2011)	No	Yes	*2a*	No	No	Yes	*sul*1, *tet*A(E)
SHY13-574	Quebec	(Trudel et al., 2016)	No	Yes	*2a*	No	Yes	Yes	No
SHY13-2627	Quebec	(Trudel et al., 2016)	No	Yes	*2a*	No	Yes	Yes	*sul*1, *tet*A(E)
SHY13-3799	Quebec	(Trudel et al., 2016)	No	Yes	*2a*	No	Yes	Yes	*sul*1, *tet*A(E)
09-0167	Quebec	(Daher et al., 2011)	Yes	No	*2a*	No	Yes	Yes	No
m15576-11	Quebec	(Emond-Rheault et al., 2015b)	Yes	No	*2a*	No	Yes	Yes	No
SHY13-2534	Quebec	(Trudel et al., 2016)	Yes	No	*2a*	No	Yes	Yes	No
07-7346	Quebec	(Daher et al., 2011)	No	No	*2a*	No	Yes	Yes	No
07-9324	Quebec	(Daher et al., 2011)	No	No	*1a*	Yes	Yes	Yes	*sul*1, *sul*2, *flo*R, *tet*A
08-2647	Quebec	(Daher et al., 2011)	No	No	*1a*	Yes	Yes	Yes	*sul*1, *sul*2, *flo*R, *tet*A
m9906-09	Quebec	(Emond-Rheault et al., 2015b)	No	No	*1a*	Yes	Yes	Yes	*sul*1, *sul*2, *flo*R, *tet*A
m9954-10	Quebec	(Emond-Rheault et al., 2015b)	No	No	*1a*	Yes	Yes	Yes	No
m15879-11	Quebec	(Emond-Rheault et al., 2015b)	No	No	*1a*	Yes	Yes	Yes	*sul*1, *sul*2, *flo*R, *tet*A
m17053-11	Quebec	(Emond-Rheault et al., 2015b)	No	No	*1a*	Yes	Yes	Yes	*sul*1, *sul*2, *flo*R, *tet*A
m23911-11	Quebec	(Emond-Rheault et al., 2015b)	No	No	*1a*	Yes	Yes	Yes	No
m17930-12	Quebec	(Emond-Rheault et al., 2015b)	No	No	*1a*	Yes	Yes	Yes	No
SHY13-3101	Quebec	(Trudel et al., 2016)	No	No	*1a*	Yes	Yes	Yes	No
SHY16-3432	Quebec	(Massicotte et al., 2019)	No	No	*1a*	Yes	Yes	Yes	*sul*1, *sul*2, *flo*R, *tet*A
SHY18-3759	Quebec	(Marcoux et al., 2021)	No	No	*1a*	Yes	Yes	Yes	*tet*A(C)
SHY13-3795	Quebec	(Trudel et al., 2016)	No	No	*1a*	Yes	Yes	Yes	*sul*1, *sul*2, *flo*R, *tet*A
AS-69-R5	Rearranged strain from m15879-11	(Paquet et al., 2019)	No	No	*1a*	Yes	No	No	*sul*1, *sul*2, *flo*R, *tet*A
890054	USA	(Gauthier et al., 2021)	No	No	*1a*	Yes	Yes	Yes	*sul*1, *tet*A(C)
01-B522	Quebec	(Daher et al., 2011)	No	No	*1a*	Yes	Yes	Yes	*sul*1, *tet*A(E)
01-B526	Quebec	(Dautremepuits et al., 2006)	No	No	*1a*	Yes	Yes	Yes	No
AS-19-R1	Rearranged strain from 08-2783	(Marcoux et al., 2021)	No	No	*1a*	Yes	No	Yes	*sul*1, *sul*2, *flo*R, *tet*A
SHY13-2317	Quebec	(Trudel et al., 2016)	Yes	No	*No*	No	Yes	Yes	No
SHY13-2825	Quebec	(Trudel et al., 2016)	Yes	No	*No*	No	Yes	Yes	No
SHY15-2816	Quebec	(Attéré et al., 2017)	Yes	No	*No*	No	Yes	Yes	No
2004-072	Canada	(Vaillancourt et al., 2021)	Yes	No	*No*	Yes	Yes	Yes	*sul*1, *sul*2, *tet*A
HER1108	Denmark	(Daher et al., 2011)	Yes	No	*1b*	Yes	No	No	No
JF3224	Switzerland	(Emond-Rheault et al., 2015a)	Yes	No	*2b*	Yes	No	Yes	No
JF3791	Switzerland	(Attéré et al., 2015)	Yes	No	*No*	Yes	No	Yes	No
JF2267	Switzerland	(Braun et al., 2002)	Intermediate	No	*No*	Yes	Yes	Yes	*sul*1*, cat*
HER1098	USA	(Daher et al., 2011)	No	No	*No*	Yes	No	No	No
HER1110	Japan	(Daher et al., 2011)	No	No	*No*	Yes	No	No	No
A449	France	(Dacanay et al., 2006)	No	No	*No*	Yes	Yes	Yes	*sul*1*, cat, tet*A(E)

a : The gene cluster correspond to gene ASA_2927 to ASA_2933 in strain A449 (Marcoux et al., 2021).

b : Type three secretion system (TTSS).

c : Only known antibiotic resistance genes to tetracycline, florfenicol, chloramphenicol and sulfonamide are indicated.

### 2.2 Phage isolation and propagation

Phage isolation was conducted according to a previously described protocol, but with slight modifications (Vincent et al., 2017). Five juvenile dead fish were obtained from a fish farm in Quebec. The mucus of each fish was carefully swabbed from the skin, fins, and gills area, and all the swabs were pooled and resuspended in 5 mL of phage buffer (50 mM Tris-HCl pH 7.5, 100 mM NaCl, 8 mM MgSO_4_) in a sterile Falcon^TM^ tube and kept at 4°C overnight. The swabs were removed, and the content was centrifuged for 10 min at 3,200 x *g* and filtered using a 0.45 μm cartridge (Sarstedt, Montreal, QC, Canada). The filtrate was then added to the same volume of 2X TSB and inoculated with 1% (v/v) of each strain (01-B516 and 2004-05 MF26) in their exponential growth phase. After overnight incubation at 18°C with agitation at 200 rpm, the cultures were centrifuged at 3,200 x *g* and filtered. The same procedure was repeated using 1X TSB until the appearance of a clear culture suggestive of phage lytic activity. Then, LB medium (Wisent, St-Bruno, QC, Canada) supplemented with 10 mM CaCl_2_ was substituted to TSB for the rest of the experiments. A volume of 100 μL of the clear culture and 400 μL overnight bacterial growth supplemented with 10 mM CaCl_2_ were plated using the double-layered agar method (Chhibber et al., 2014). Phage plaques were observed with both *A. salmonicida* subsp. *salmonicida* strains (01-B516 and 2004-05 MF26). The rest of the experiments were continued using strain 2004-05 MF26 as a host because phage plaques were larger.

Ten plaques were randomly selected with sterile truncated pipette tips, and each plaque was diffused in 500 μL of phage buffer for 30 minutes. A volume of 100 μL of each diffused plaque and 400 μL overnight bacteria were plated according to the double-layered agar method, in presence of 10 mM CaCl_2_. This step was repeated 3 times for phage purification purposes. After purification and propagation of each 10 plaques, 25 μl of each phage lysate was mixed in 25 μl of SWL buffer (50 mM KCl, 10 mM Tris, pH 8.3, 2.5 mM MgCl_2_, 0.45% NP-40, and 0.45% Tween 20) (Charette and Cosson, 2004). Then, all samples were heated up to 95°C for 5 min for preparation of DNA template for randomly amplified polymorphic DNA (RAPD) using primers #18 (5’- GCCAGCAGG-3’) and #20 (5’- GCCAGCAGC-3’). Each PCR master mix contained a final concentration of 1X Go-Taq buffer (Promega, USA), 1.9 μl of 0.2 mM dNTP, 60 μM of RAPD primers (#18 or #20), 1.25 U of GoTaq^TM^ DNA polymerase, 1 μl of DNA template in a final volume of 20 μL completed with nuclease-Free water. The PCR program was set at 95°C for 5 min, following by 40 cycles of 30 s at 95°C, 30 s at 37°C and 1 min at 72°C, with a final extension at 72°C for 10 min. Samples were run on 1% agarose gel during 70 min at 100 V, followed by ethidium bromide staining for 30 min (0.5 μg/ml) and then in a water bath for 1 h before being visualized under UV (Gutiérrez et al., 2011). PCR reactions were done twice, and many controls were added to validate the experiment. The selected phage MQM1 was propagated by plate lysate method, and chloroform-treated to obtain clear lysate with a phage titer of >10^9^ pfu/mL (Bonilla et al., 2016; Su et al., 1998). The phage lysate was stored with 15% glycerol at -70°C or kept at 4°C until use within a period of 2 months.

### 2.3 Phage host range determination using efficiency of plating

The host range of phage MQM1 was determined on 60 additional *A. salmonicida* subsp. *salmonicida* isolates with diverse genetic profiles (Table 1), and on 45 mesophilic and psychrophilic bacteria belonging to 35 different species or subspecies (representing 10 bacterial genera, Table S1). In the case of *A. salmonicida* subsp. *salmonicida* strains, they were selected in order to have a representation of different characteristics such as the presence or absence of *i)* an A-layer (a protein layer on the surface of the cells), *ii)* the type three secretion system (TTSS, a major virulence factor) or *iii)* a gene cluster (ASA_2927 to ASA_2933) associated with the instability of the plasmid pAsa5 and which may be linked to a prophage (Dallaire-Dufresne et al., 2014; Marcoux et al., 2021). The efficiency of plating (EOP) procedure used was described elsewhere (Leduc et al., 2021; Vincent et al., 2017) with slight modifications. Psychrophilic isolates were thawed and streaked on TSA plates and incubated at 18°C for 3 days, then 2 to 5 colonies were inoculated in LB broth and incubated overnight at 18°C with agitation at 200 rpm. For each strain, a volume of 400 µL overnight bacterial growth was mixed to 3 mL of soft 0.75% LB agar kept at 55°C and layered on thin LB agar. Since plaque size increased in presence of CaCl_2_ in the culture medium, all media were supplemented with 10 mM CaCl_2_. The phage stock was serially diluted, and 10 or 15 µL of each 1/10 dilution were directly deposited onto the inoculated plates, let to dry, and incubated overnight at 18°C. The same protocol was used for mesophilic strains, except that they were incubated at 25°C. The EOP experiments were performed at least twice for each strain.

### 2.4 Phage lytic efficacy in liquid assay

The growth curves of strains 01-B516 and 2004-05 MF26 were determined in LB in absence or in presence of phages at three initial MOI values of 0.1, 1, and 10, with a protocol previously described (Hosseini et al., 2021). In addition to highly-sensitive strains, the low-sensitive strains were also tested in liquid assay. The incubation period was 30 h at 18°C, with agitation and the experiments were done in biological duplicates.

### 2.5 Phage adsorption assay

Strain 2004-05 MF26 carrying *AsaGEI2a* and Prophage 3 as well as strain 09-0167 with only *AsaGEI2a* (Table 1) were chosen to investigate the adsorption of phage MQM1. The bacterial strains were grown separately in 10 mL fresh LB with 10 mM CaCl_2_ until they reached an optical density (OD) of 0.6 to 0.8 at 600 nm. A volume of 100 μL of diluted phage suspension (down to 1500 pfu/mL) was added to 900 μL of the bacterial strain. Several 1.5 mL microtubes were prepared for consecutive phage/bacteria incubation times (every 5 min for 25 min). For the control, the same volume of phages was added to 900 μL fresh LB with 10 mM CaCl_2_ and tubes were incubated at 18°C in a tube rotator. At each time point, the host strain and the control tube were centrifuged at V_max_ for 1 min and 100 μL of supernatants were tittered by double-layer agar method in triplicate. The phage adsorption was calculated as reported elsewhere (Duplessis and Moineau, 2001).

### 2.6 Genomic DNA extraction and genomic sequencing

A high-titer phage lysate (>10^9^ pfu/mL) was prepared using the plate lysate method. Phage genomic DNA was extracted using the DNeasy blood and tissue kit (Qiagen, Montreal, QC, Canada) (Jakočiūnė and Moodley, 2018). Sequencing libraries were then prepared using NEBNext® Ultra™ II. The sequencing was performed using a MiSeq system (Illumina, San Diego, CA, USA). A total of 122,861 paired-end reads were generated at the Plateforme d'Analyse Génomique of the Institut de Biologie Intégrative et des Systèmes (IBIS) at Université Laval.

### 2.7 Genome assembly, analysis, and annotation

The read quality was analyzed using FastQC (version 0.11.9, https://www.bioinformatics.babraham.ac.uk/projects/fastqc/). The *de novo* assembly was done using Shovill version 1.1.0, alongside the activation of module trimmomatic version 0.39 to remove the Illumina adaptors, and pilon version 1.24 was used to help polishing the reads. The estimated sequencing depth was 739x, which was automatically subsampled to 150x by the assembler. The whole reads were mapped to the assembled contig using BWA version 0.7.17-r1188 (Li, 2013) and SAMtools version 1.10 (Li et al., 2009). The assembled contig was further visually assessed with bandage version 0.9.0 (Wick et al., 2015). The subsequent single-contig was confirmed by PCR using primers listed in Table S2. The ORF prediction was done using Metageneannotator (Galaxy version 1.0.0) and Web Apollo interfaces (https://cpt.tamu.edu/galaxy-pub). The sequence annotation was done using RAST version 2.0 (Aziz et al., 2008), Prokka v1.14.5 (Seemann, 2014), and DFAST version 1.2.18 (Tanizawa et al., 2018), and the output files were visualized, compared, and verified using Artemis version 18.1.0 (Carver et al., 2012). The coding sequences were then analyzed with the help of Blastp (https://blast.ncbi.nlm.nih.gov/Blast.cgi) and HHpred (https://toolkit.tuebingen.mpg.de/tools/hhpred) (Zimmermenn et al., 2018). The conserved protein domains of each predicted ORF were analysed through https://www.ncbi.nlm.nih.gov/Structure/cdd/wrpsb.cgi. The tRNA prediction was done using Aragorn http://www.ansikte.se/ARAGORN/ (Laslett and Canback, 2004) and tRNAscan-SE 2.0 (http://lowelab.ucsc.edu/tRNAscan-SE/) (Chan et al., 2021; Lowe and Eddy, 1996). The selection settings were as described at https://seaphages.org/media/docs/Predicting_tRNA_and_tmRNA_genes_12-2-16.pdf. The genomic map was created by EasyFig version 2.2.2 (Sullivan et al., 2011), and the gene cluster comparisons with the most similar phage was done using Clinker version 0.0.23 (Gilchrist and Chooi 2021). PhageTerm algorithm (Galaxy Version 1.0.11) (Ramsey et al., 2020) was used for the computational determination of phage termini (Garneau et al., 2017). ABRicate (version 1.0.1) was used to search for probable antibiotic-resistant genes through https://usegalaxy.org. The viral proteomic tree server (ViPTree version 3.5) available at https://www.genome.jp/viptree was used for global genomic comparison with other viruses (Nishimura et al., 2017). VIRFAM algorithm was used for structural gene analysis based on head-neck-tail module of previously identified tailed phages (Lopes et al., 2014). BACPHLIP (Galaxy version 1.0) was used for lifestyle prediction (Hockenberry and Wilke, 2021). The complete and annotated phage genome is available at NCBI GenBank under the accession number OQ628262.

### 2.8 Transmission electron microscopy (TEM)

Phage MQM1 was observed with a transmission electron microscope (TEM) as previously described (Vincent et al., 2017) with slight modifications. Phage lysate (4 mL) was centrifuged at 23,500 × *g* for 1 hour at 4°C and the subsequent pellet was washed twice with ammonium acetate (0.1 M, pH 7.0). The concentrated phage lysate was added to the formvar/carbon supported nickel grids (Sigma-Aldrich, Oakville, ON, Canada) and stained with 2% phosphotungstic acid pH 7.0 for 2 min or with 2% uranyl acetate for 5 min. At least 10 images were taken with a JEOL 1230 TEM at the Imaging - Microscopy Platform of the IBIS at Université Laval. Images were uploaded on ImageJ software version 1.53t to estimate the phage dimension (Schneider et al., 2010).

## 3 Results and discussion

### 3.1 Phage isolation and propagation

Phage vB_AsaP_MQM1 was isolated from the mucus of five juvenile fish obtained from a fish farm located in the southern part of Quebec. A furunculosis outbreak was unusual at that time of the year. The fish were obtained dead from the fish farm without an official diagnosis of furunculosis for that particular fish, but the fish farm was experiencing an outbreak of furunculosis being treated by a veterinarian at the same time. The samples were gathered on October 28^th^, 2021. The maximum air temperature at this time was below 10°C.

**Fig. 1:**
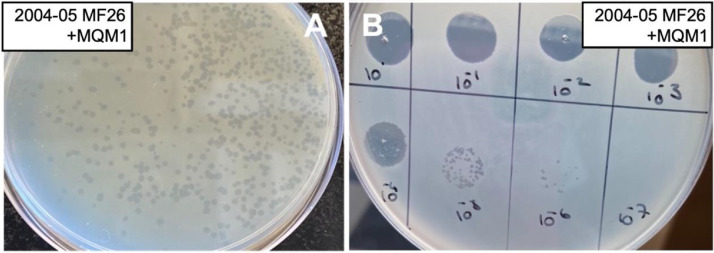
**MQM1 is a lytic phage, and its host is *A. salmonicida* subsp. *salmonicida* 2004-05 MF26, which bears Prophage 3. A.** Lytic plaques produced by phage MQM1 on a bacterial lawn of strain 2004-05 MF26 grown on LB agar supplemented with 10 mM CaCl_2_. **B.** EOP of phage MQM1 on 2004-05 MF26.

During the initial steps of phage isolation from the mucus sample, when it was incubated with a bacterial host bearing Prophage 3, the liquid medium was clearer in comparison to the growth of the bacterial host alone, suggesting the presence of lytic phages. Phage plaques were readily observed with both Prophage 3-bearing strains 01-B516 and 2004-05 MF26 in the doubled-layer agar method. The plaque size was larger, between 0.5 and 2 mm, in the presence of 10 mM of CaCl_2_ in the culture medium (Fig. 1), particularly with the strain 2004-05 MF26, which was then chosen as the amplification host for the remaining experiments. Ten phage plaques were analyzed by RAPD PCR. All the samples showed a similar pattern on agarose gel (Fig. S1) suggesting the presence of one specific phage. One of the corresponding plaques was randomly chosen and was named MQM1 and further characterized.

A chloroform treatment of phage MQM1 lysate showed no decrease in phage titer (Bonilla et al., 2016), likely indicative of a protein-only nature of the virion structure. However, the titer of the phage lysate decreased by more than 2 logs within a week at room temperature but not at 4°C.

### 3.2 MQM1 host range

**Fig. 2:**
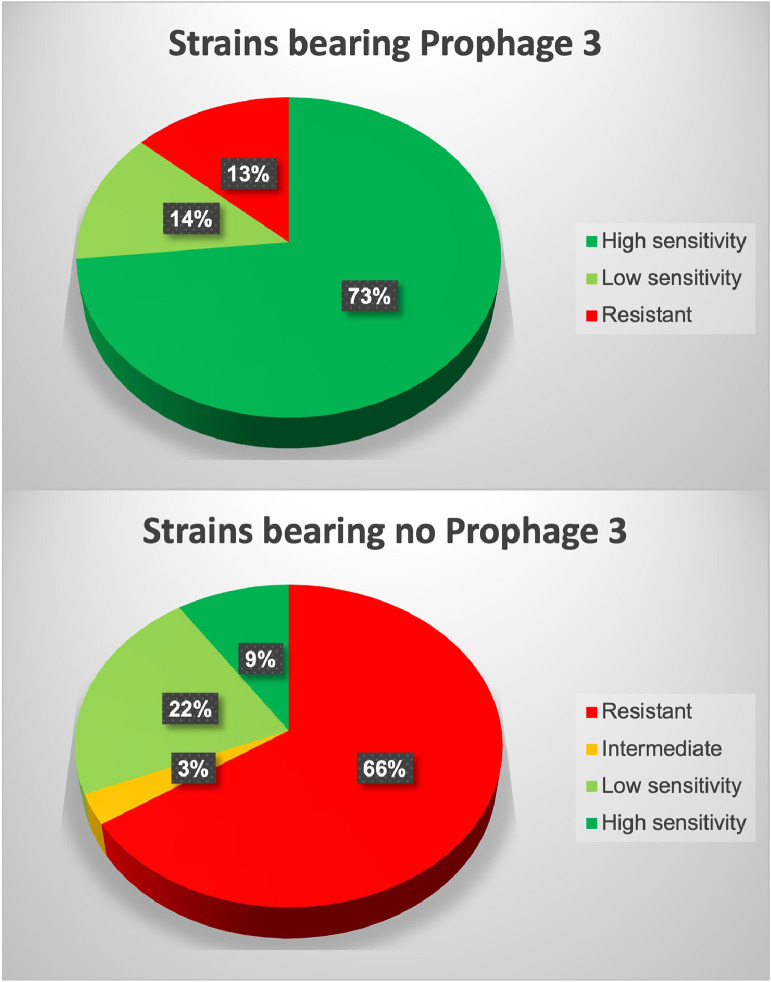
**Host range of phage MQM1 on bacterial strains with and without Prophage 3**. These graphs include all the strains found in Table 1 and Table S1 and were classified in two groups depending on the presence (*n*=30, in **A**) and absence (*n*=32, in **B**) of Prophage 3.

Since phage MQM1 infects strains 01-B516 and 2004-05 MF26 which harbor Prophage 3, we determined its host range and assess its ability to infect other strains carrying Prophage 3 as well as strains without it. The EOP of MQM1 was performed with 45 mesophilic and psychrophilic bacteria belonging to 35 different bacterial species and subspecies (Fig. 2, Tables 1 and S1). Our data showed that this phage is specific to *A. salmonicida* subsp. *salmonicida* strains, with two exceptions: 1) strain *A. salmonicida* M18076-11, which is an atypical *A. salmonicida* subspecies isolated from a lumpfish in North America producing unusual granular structures was highly sensitive to MQM1; and 2) strain *A. salmonicida* subsp. *achromogenes* JF2997 was sensitive to MQM1 while the three other strains tested from the *achromogenes* subspecies were resistant.

The EOP results also showed that within the *salmonicida* subspecies, out of the 62 strains, 36 strains were completely lysed by phage MQM1 (Table 1). Among MQM1-sensitive strains, most of them contain Prophage 3. In fact, out of 30 Prophage 3-carrying strains, 26 strains were sensitive to phage MQM1: 22 strains were highly sensitive (phage plaques obtained with phage dilutions range between 10^4^ -10^6^) and four strains were slightly sensitive (phage plaques obtained with phage dilutions between 10^1^ – 10^3^ pfu/mL). Generally, phages used for phage therapy are classified in three categories: 1) a phage with narrow host range which infects only some strains of the same species; 2) a phage with broader host range which infects several strains, and 3) a polyvalent host range phage with a host spectrum that goes beyond one species (Hyman, 2019). MQM1 could be classified in the second category, since it infects a large range of strains from only one subspecies (with the two exceptions mentioned above).

The isolation of this new phage, which is capable of infecting strains harboring Prophage 3, was mandatory to improve the previously designed cocktail against furunculosis (Hosseini et al., 2021). It is interesting that the infectivity of this phage is not limited to Prophage 3-bearing strains. Out of the 30 *A. salmonicida* subsp. *salmonicida* strains tested without Prophage 3, 11 strains were sensitive to various degrees (Table 1). Due to our knowledge of the genetic profile of *A. salmonicida* subsp. *salmonicida* strains tested in this study, we noticed that with a few exceptions, strains possessing a specific gene cluster (ASA_2927 to ASA_2933) (Marcoux et al., 2021) were generally resistant to phage MQM1 (Table 1). This observation is worth noting because out of 174 Prophage 3 positive strains in our *A. salmonicida* subsp. *salmonicida* collection, only 2 strains have this gene cluster (data not shown). It appears that in most cases, Prophage 3 and this gene cluster are mutually exclusive in a given strain. Since we previously have isolated (myo)phages capable of infecting strains carrying this gene cluster (Hosseini et al., 2021; Vincent et al., 2017), the availability now of a phage that can bypass Prophage 3 protection may improve our phage cocktail against *A. salmonicida* subsp. *salmonicida*.

### 3.3 Phage lytic efficacy in liquid assay

**Fig. 3:**
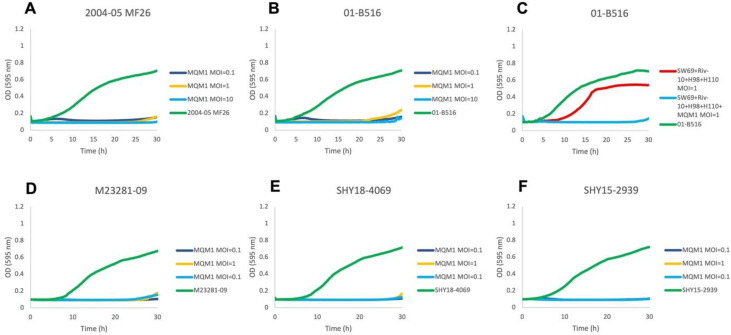
**Phage MQM1 inhibits the growth of bacteria insensitive to a previously designed phage cocktail.** Growth curves of *A. salmonicida* subsp. *salmonicida* strain 2004-05 MF26 (**A**) and 01-B516 bearing Prophage 3 (**B**) for 30 h alone and in presence of various initial MOIs of phage MQM1. **C.** Action of the previously designed phage cocktail on the growth of strain 01-B516 for 30 h in the presence and absence of phage MQM1. Growth curves of *A. salmonicida* subsp. *salmonicida* strains M23281-09 **(D)**, SHY18-4069 **(E)** and SHY15-2939 **(F)** bearing Prophage 3 and having a low sensitive effect according to the EOP results. They were grown for 30 h at 18°C alone and in presence of various initial MOIs of phage MQM1.

The myophages previously included in the phage cocktail demonstrated lytic activity against strains having Prophage 3 in the EOP on solid media, but they had a lower effect on these same bacterial strains when tested in liquid culture (Hosseini et al., 2021). We therefore tested the lytic activity of phage MQM1 in liquid culture. According to growth curves of both strains 01-B516 and 2004-05 MF26, phage MQM1 was able to completely inhibit them for more than 24h at three initial MOI values: 0.1, 1, and 10 (Fig.s 3A and B), including when part of the phage cocktail (Fig. 3C). Similar results were also obtained for the three *A. salmonicida* subsp. *salmonicida* strains that showed low-sensitivity to MQM1 in EOP (Fig. 3D, E & F). These results suggest that phage MQM1 could represent a significant addition in a cocktail of phages to control *A. salmonicida* subsp. *salmonicida*. However, MQM1 is more efficient in the presence of calcium. It will therefore be important to evaluate the effectiveness of phage MQM1 in real aquaculture conditions with variable concentrations of calcium.

### 3.4 Adsorption assay

**Fig. 4:**
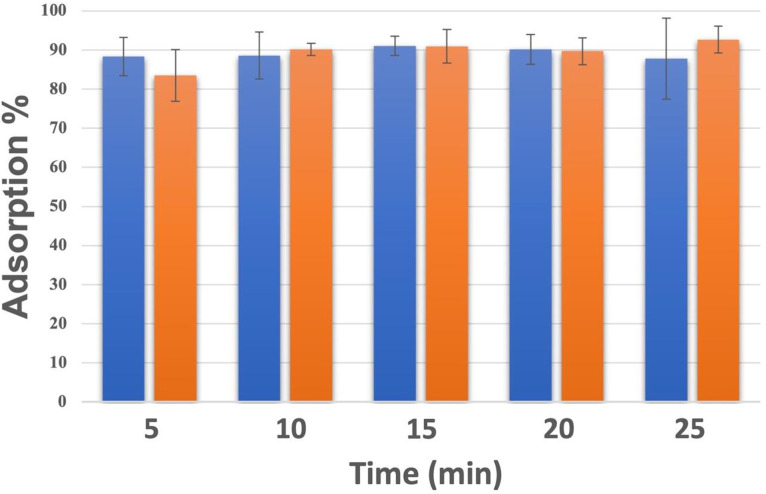
**The adsorption rate of phage MQM1.** The adsorption rates of phage MQM1 on *A. salmonicida* subsp. *salmonicida* strain 09-0167 without Prophage 3 (blue) and on strain 2004-05 MF26 with Prophage 3 (orange) are shown.

Since phage MQM1 was very efficient in the liquid assay, we were interested in its adsorption kinetics. We investigated phage adsorption using strain 2004-05 MF26, which is the host of MQM1, and another sensitive strain, namely strain 09-0167, (Table 1). The results of the adsorption assays are shown in Fig. 4. After only 5 min of contact between the phages and bacterial cells, 83.3 ± 6.6 % of phage particles were attached to the surface of strain 2004-05 MF26, which carries *AsaGEI2a* and Prophage 3. This adsorption rate increased to 92.6 ± 3.4 % after 25 min. For strain 09-0167 with only *AsaGEI2a*, close to 88% of phage particles attached to the surface after 5 min and remained mostly stable over time.

Previously, we analyzed the adsorption kinetics of the myophage SW69-9 on its host *A. salmonicida* subsp. *salmonicida* M15879-11, which has the A-layer on its surface, as well as with mutant derivatives without the A-layer (Paquet et al., 2019). The adsorption rate of phage SW69-9 was much lower, i.e. 15% after 5 min, but more than 70% with the mutant deficient in A-layer. Further characterization of phage SW69-9 led to the discovery that its receptor was a part of the lipopolysaccharides (LPSs), which was likely hidden by the A-layer. Here, our results shown in Table 1 suggest that phage MQM1 receptor is not linked to the A-layer because two strains (57043 and HER1108) without an A-layer are also sensitive to phage MQM1.

### 3.5 MQM1 genome and morphology

Before considering the use of a phage for therapeutic applications, it is essential to confirm that its genome does not contain genes that may enable the host bacterium to acquire genes contributing to its virulence or its ability to resist antibiotics. Consequently, the MQM1 genome was sequenced and analyzed.

**Fig. 5:**
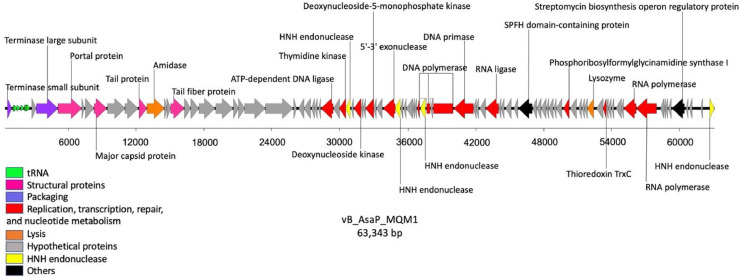
**Genomic map of phage MQM1**. Genes are represented by arrows. The general function deduced from the product of these genes is color-coded as indicated in the legend. The predicted functions to each gene are based on the best matches on Blastp, HHpred, and NCBI conserved domain.

The assembly and trimming yielded a single contig of 63,343 bp, linear dsDNA (Fig. 5). The GC content of MQM1 genome (50.2%) is lower that its host *A. salmonicida* subsp. *salmonicida,* (58.5%), and the number of coding sequences (CDS) was predicted to be 88 (Table S3). The genome of phage MQM1 also possesses 8 tRNA (Table S4) as predicted by ARAGORN and supported by tRNAscan-SE output. The GC content of the tRNA ranges from 49.3% to 65.3%. (Table S4). No antibiotic-resistant genes were found in MQM1 genome according to ABRicate and the Bacterial and Viral Bioinformatics Resource Center (BV-BRC) output results. Also, according to BACPHLIP, the lifestyle of the phage is predicted to be virulent, which is compatible with the culture assays and the absence of an integrase gene in its genome (Table S3).

Since the phage genome was sequenced using the random fragmentation method for library preparation, the PhageTerm method was used to determine the phage termini (Garneau et al., 2017). The PhageTerm analysis, which is based on the biases in the number of reads at termini location, predicted two direct terminal repeats (DTR) of 327 bp. Therefore, the *in silico* packaging mechanism predicted for this phage is similar to the podophage T7, with short terminal repeats (Garneau et al., 2017).

**Fig. 6:**
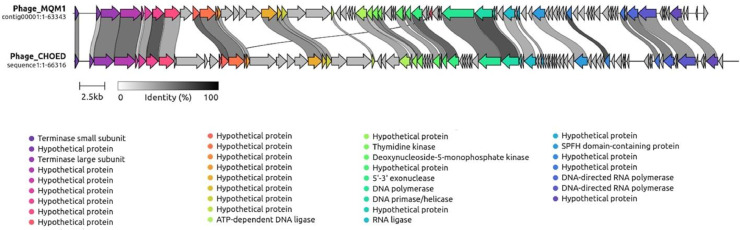
**A representation of the genomic alignment of Aeromonas phage MQM1 with the genome of Vibrio phage CHOED**. This analysis was performed using Clinker. This software identified similar gene clusters between two phage genomes. The attributed function of each gene is identified by the same color. High identity regions are shown in shades of grey.

**Fig. 7:**
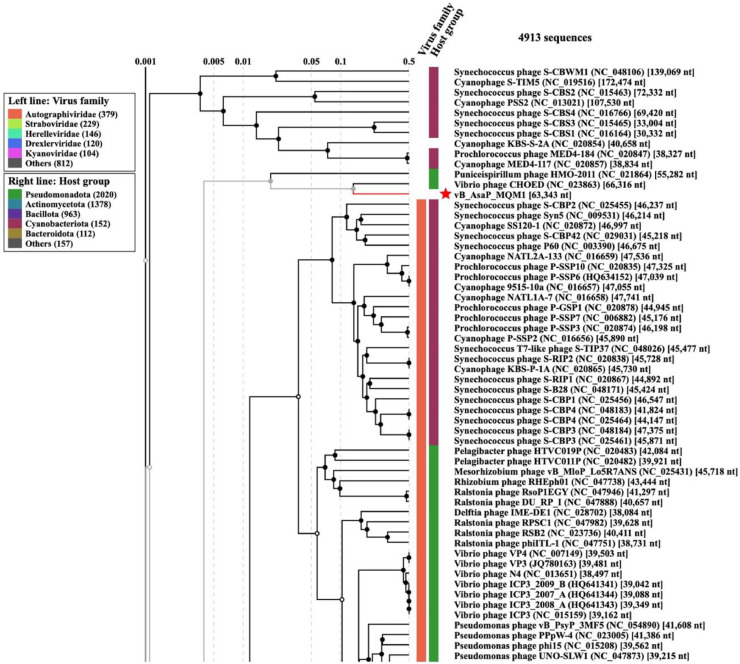
**The extended view of ViPTree analysis for phage MQM1.** The phylogenetic tree was constructed using 4913 phage sequences as reference using ViPTree version 3.5. The closest clade to phage MQM1 (red branch, shown by red star) is Vibrio phage CHOED. Only a part of the phylogenetic three is shown in this figure.

Comparative genomic analyses (Blastp) showed that the closest relative to phage MQM1 is *Vibrio* podophage CHOED (Romero et al., 2014) (accession number: KJ192399.2), with a percentage of identity of 67.3% (Fig. 6). ViPTree analysis also demonstrated that phage MQM1 is close to phage CHOED (Fig. 7). VIRFAM results showed that the capsid and the connector organization of phage MQM1 fit the type 3 neck category. Typically, in a podophage of type 3, the general gene organization is [TermL-x_(0-3)_-Portal-x_(1-4)_-MCP-x_(1-5)_-Ad3-x_(0-3)_-Hc3] which corresponds to the architecture of phage MQM1 (Lopes et al., 2014) (Fig. S2). The annotation predicted three adjacent ORFs containing DNA polymerase I superfamily conserved domain (cl02626) and the closest match to all these ORFs was DNA polymerase I of Vibrio phage CHOED. However, the Vibrio phage CHOED DNA polymerase I has 747 aa, while the size of these three ORFs predicted to contain DNA polymerase I conserved domain in phage MQM1 are 590 aa, 115 aa, and 56 aa, respectively. These three ORFs in phage MQM1 are intervened by two other ORFs. One of the two intervening ORFs is predicted to encode for protein with HNHc superfamily domain (e-value= 7.72e-13), and the closest match to this ORF is Vibrio phage CHOED HNH endonuclease with the accession number of YP_009021706.1 (69.7 identity, 92% coverage, and e-value of 5e-62). In Vibrio phage CHOED, the gene coding for an HNH endonuclease is located at the upstream of DNA polymerase I. In bacterial or phage genomes, sometimes a coding region can be interrupted by a self-splicing intron or intein. These introns can contain an internal ORF, encoding mobility-promoting homing-endonucleases (Friedrich et al., 2007). Homing endonucleases have been described as mobile sequences within intron group I (Chevalier and Stoddard, 2001). The presence of a predicted ORF encoding HNH endonuclease which have disrupted the DNA polymerase gene in MQM1 phage suggests the presence of putative introns.

**Fig. 8:**
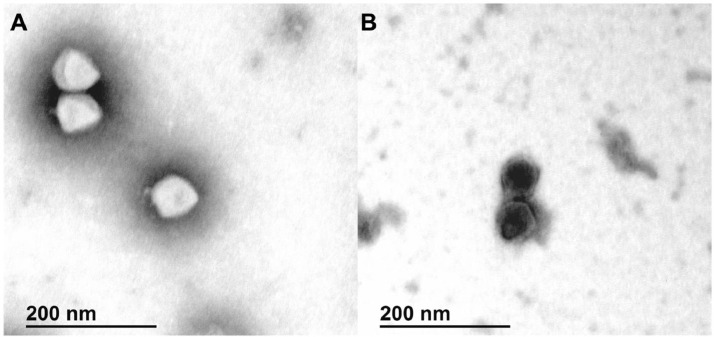
**TEM micrograph of phage MQM1 virions. A.** Virions were stained with 2% phosphotungstic acid and **B.** with 2% uranyl acetate.

According to TEM micrographs, phage MQM1 has an icosahedral capsid and a short tail (Fig. 8). The average diameter of the capsid is 70.6 ± 1.8 nm, and the tail is 9.2 ± 1.9 nm in length. Therefore, MQM1 shows a typical podophage morphology, which is compatible with our results from ViPtree analysis. According to Bai *et al.,* only one other podophage (PhiAS7) specific to *A. salmonicida* was found in genomic databases among more than 25 characterized phages known to have *Aeromonas* genus as host (Bai et al., 2019). PhiAS7, a T7-like member of the *Autographiviridae* family, was found in a sediment from a fish farm in Korea. It seems that this podophage has a very narrow host range as it was limited to two *A. salmonicida* subsp. *salmonicida* strains from those tested (Kim et al., 2012). Another podophage capable of infecting *A. salmonicida* has been recently described, namely phage T7-Ah (HER212). This phage was isolated from an unknown sample from Spain and infects some mesophilic *A. salmonicida* strains as well as *A. salmonicida* subsp. *acchromogenes* strains but not strains from the *salmonicida* subspecies (Leduc et al., 2021). It is important to mention that no similarity was observed between the genomic sequence of phages MQM1 and PhiAS7 (accession: NC_019528.1) or between MQM1 and T7-Ah (accession: MT740748.1) even though these three podophages infect *A. salmonicida,* indicating that they belong to different taxonomic groups.

## 4 Conclusion

Generally, there are two approaches for phage therapy: “sur mesure” which means a custom-designed to each case and “prêt-à-porter” where you have a one-size-fits-all general approach. The first personalized method consists of selecting and applying specific phages to kill the pathogen isolated from the patient or the environment. Meanwhile, the purpose of the second method is to create a ready-to-use phage cocktail with multiple phages with a broad host range, capable of lysing pathogens that are commonly found in a specific infection (Smug et al., 2022). In both cases, the presence of phage resistance mechanisms that interfere with phage therapy is a concern. Our purpose was to improve a phage cocktail in pursuit of a ready-to-use cocktail for aquaculture applications and tailored to *A. salmonicida* subsp. *salmonicida*. The discovery and *in vitro* characterization of phage MQM1 indicated that this phage offers potential for eliminating strains of *A. salmonicida* subsp. *salmonicida* that bear Prophage 3. The latter usually provides resistance against several phages through a currently unknown defense mechanism(s). To our knowledge, MQM1 is only the second podophage that has lytic activity against *A. salmonicida* subsp. *salmonicida* strains and the first outside Asia. This new bacterial virus adds to our arsenal of virulent phages that may be eventually used in fish farms to combat furunculosis.

## CRediT authorship contribution statement

**Nava Hosseini:** Conceptualization, Data curation, Formal analysis, Investigation, Methodology, Resources, Software, Validation, Visualization, Writing – original draft, Writing – review & editing. **Valérie E. Paquet:** Formal analysis, Investigation, Methodology, Resources, Validation, Writing – original draft, Writing – review & editing. **Pierre-Étienne Marcoux:** Data curation, Formal analysis, Investigation, Software, Validation, Visualization, Writing – review & editing. **Charles-Antoine Alain:** Investigation, Writing – review & editing. **Maude F. Paquet:** Investigation, Writing – review & editing. **Sylvain Moineau:** Funding acquisition, Supervision, Validation, Writing – review & editing. **Steve J. Charette:** Conceptualization, Formal analysis, Funding acquisition, Methodology, Project administration, Supervision, Validation, Visualization, Writing – original draft, Writing – review & editing.

## Declaration of Competing Interest

The authors declare that they have no known competing financial interests or personal relationships that could have appeared to influence the work reported in this paper.

## Appendix Supplementary materials

Image, application 1
