# Supplementary material for: MQM1, a bacteriophage infecting strains of Aeromonas salmonicida subspecies salmonicida carrying Prophage 3
Source: Virus Res. 2023 Jun 30;334:199165. doi: 10.1016/j.virusres.2023.199165 (PMC10410586; doi:10.1016/j.virusres.2023.199165)
Supplement: Supplementary file 1 [file mmc1.docx]

*Supplementary data*

**MQM1, a bacteriophage infecting strains of *Aeromonas* *salmonicida* subspecies *salmonicida* carrying Prophage 3**

Nava Hosseini^1,2,^*, Valérie E. Paquet^1,2,3^, Pierre-Étienne Marcoux^1,2^, Charles-Antoine Alain^1,2^, Maude F. Paquet^1,2^, Sylvain Moineau^1,2,4,5^ and Steve J. Charette^1,2,3,^*

1. Institut de Biologie Intégrative et des Systèmes (IBIS), Pavillon Charles-Eugène-Marchand, Université Laval, Quebec City, QC, G1V 0A6, Canada.

2. Département de biochimie, de microbiologie et de bio-informatique, Faculté des sciences et de génie, Université Laval, Quebec City, QC, G1V 0A6, Canada.

3. Centre de Recherche de l’Institut Universitaire de Cardiologie et de Pneumologie de Québec (IUCPQ), Quebec City, QC, G1V 4G5, Canada.

4. Groupe de Recherche en Écologie Buccale (GREB), Faculté de médecine dentaire, Université Laval, Quebec City, QC, G1V 0A6, Canada.

5. Félix d’Hérelle Reference Center for Bacterial Viruses, Université Laval, Quebec City, QC, G1V 0A6, Canada.

* Correspondence: Institut de Biologie Intégrative et des Systèmes (IBIS), Pavillon Charles-Eugène-Marchand, Université Laval, Quebec City, QC, G1V 0A6, Canada [nava.hosseini.1@ulaval.ca](mailto:nava.hosseini.1@ulaval.ca), [steve.charette@bcm.ulaval.ca](mailto:steve.charette@bcm.ulaval.ca).


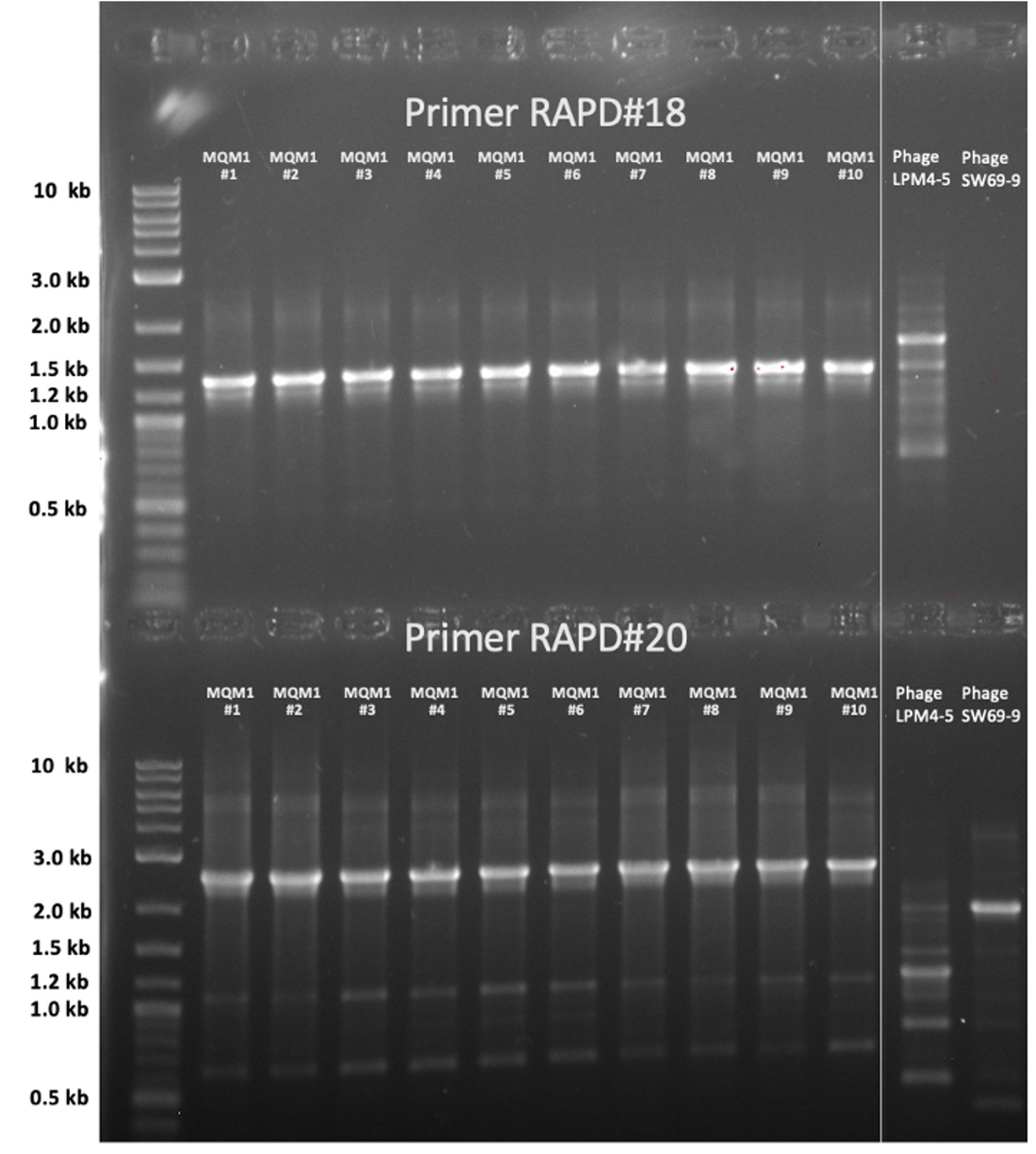


**Figure S1. RAPD PCR profile of 10 phage isolates using two RAPD primers (#18 and #20).** As controls, two different phages (myophages) from our collection including phage LPM4-5 and SW69-9 were used. The profiles of the 10 isolates suggested that they were genetically highly similar.


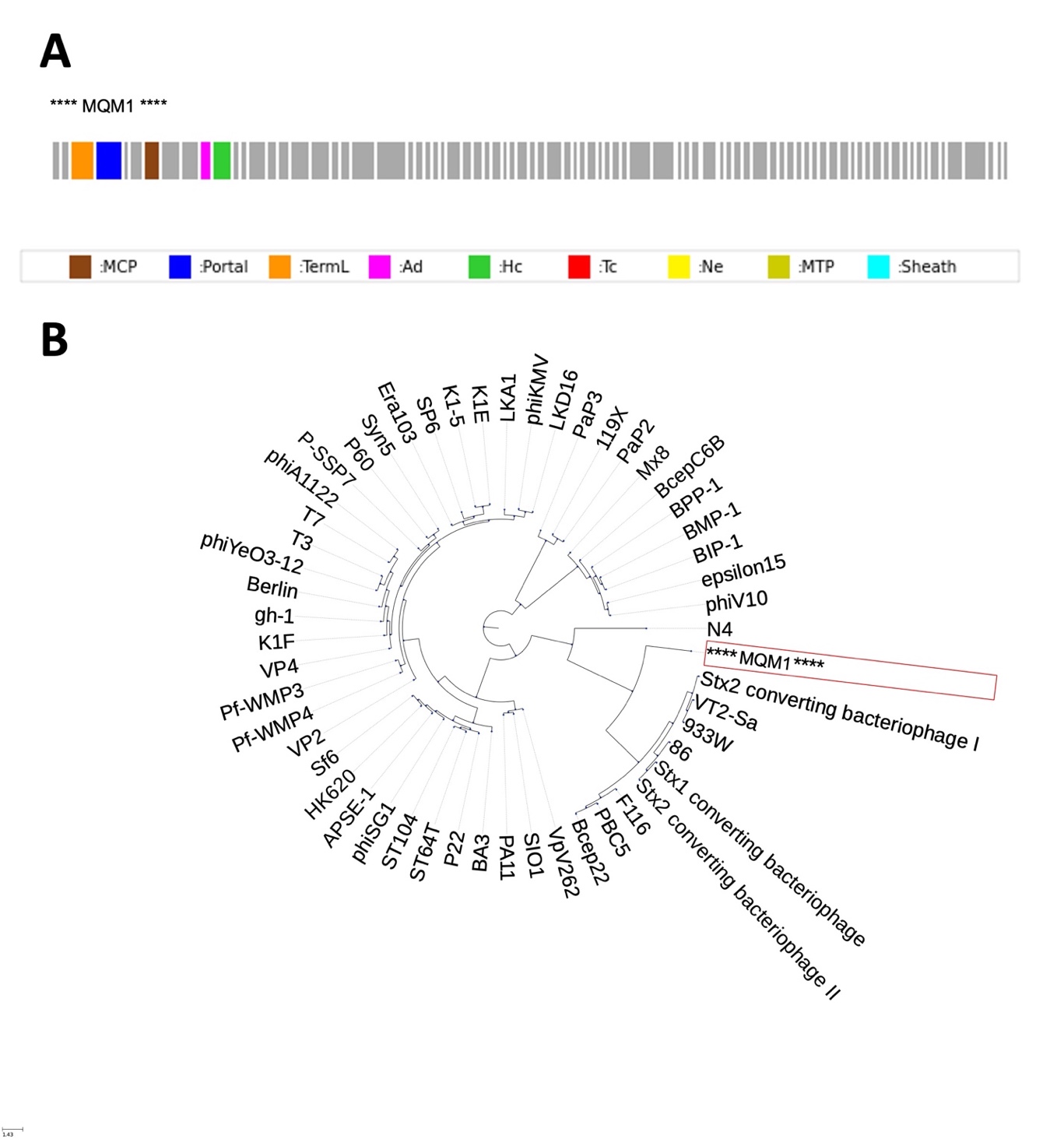


**Figure S2. Classification of phage MQM1 based on VIRFAM analysis.** The VIRFAM algorithm performs an analysis based on the head-neck-tail module of known tailed phages (Lopes et al., 2014). This computational program is designed on the fact that only limited modules are used by phages to create their virion structure. **A.** Graphical representation of the components identified in the genome of MQM1. Rectangles represent the genes of MQM1 and those with colors are coding for the corresponding proteins in the legend (MCP: Major Capsid Protein, Portal: Portal protein, TermL: large terminase subunit, Ad: Adaptor, Hc: Head closure, TC: Tail Completion, Ne: Ne1 protein, MTP: Major Tail Protein, Sheath: Sheath protein). **B.** Clustering of the query phage with respect to those of the same type. MQM1 is categorised as Type 3 podophage and falls within the same large group as phage T7 and several other podophages.

**Table S1. Host range of phage MQM1 on bacterial species and subspecies other than *A. salmonicida* subsp. *salmonicida*.**

| Bacteria | Sensitivity to phage MQM1 | Reference or source* |
| --- | --- | --- |
| *A. salmonicida* subsp. *salmonicida* 2004-05 MF26 (MQM1 host) | Yes | (Vincent et al., 2014) |
| *A. salmonicida* M18076-11 | Yes | (Rouleau et al., 2018) |
| *A. salmonicida* subsp. *achromogenes* JF2997 | Intermediate | (Burr et al., 2005) |
| *A. salmonicida* subsp. *achromogenes* JF3115 | No | (Studer et al., 2013) |
| *A. salmonicida* subsp. *achromogenes* JF3116 | No | (Burr et al., 2005) |
| *A. salmonicida* subsp. *achromogenes* JF3499 | No | (Burr and Frey, 2007) |
| *A. salmonicida* subsp. *masoucida* JF3118 | No | (Burr et al., 2005) |
| *A. salmonicida* subsp. *pectinolytica* JF3120 | No | (Pavan et al., 2000) |
| *A. salmonicida* subsp. *smithia* JF4097 | No | (Goldschmidt-Clermont et al., 2009) |
| *A. salmonicida* subsp. *smithia* JF4460 | No | (Goldschmidt-Clermont et al., 2009) |
| *A. salmonicida* A15-3132 | No | This study |
| *A. salmonicida* A527 | No | (Nagar et al., 2011) |
| *A. salmonicida* HER1209 | No | FHRC |
| *A. salmonicida* JF2480 | No | (Vincent et al., 2019) |
| *A. salmonicida* K | No | (Nikapitiya et al., 2019) |
| *A. salmonicida* Y47 | No | (Nagar et al., 2011) |
| *A. salmonicida* Y528 | No | (Nagar et al., 2011) |
| *A. salmonicida* Y559 | No | (Nagar et al., 2011) |
| *A. salmonicida* Y567 | No | (Nagar et al., 2011) |
| *A. salmonicida* Y577 | No | (Nagar et al., 2011) |
| *A. salmonicida* sp. 19-K304 | No | (Attéré et al., 2023) |
| *A. salmonicida* sp. 19-K308 | No | (Attéré et al., 2023) |
| *Aeromonas hydrophila* HER1210 | No | FHRC |
| *Aeromonas hydrophila* HER1214 | No | FHRC |
| *Aeromonas molluscorum* JF3328 | No | (Miñana-Galbis et al., 2004) |
| *Aeromonas sobria* JF2635 | No | (Gauthier et al., 2017b) |
| *Aeromonas veronii* JF3071 | No | (Küpfer et al., 2006) |
| *Aeromonas veronii* biovar *sobria* HER1216 | No | FHRC |
| *Bacillus cereus* | No | (Berthiaume et al., 2014) |
| *Cupriavidus* sp. isolate 1 | No | (Berthiaume et al., 2014) |
| *Cupriavidus* sp. isolate 2 | No | (Berthiaume et al., 2014) |
| *Delftia* sp. | No | (Berthiaume et al., 2014) |
| *Escherichia coli* HER1024 | No | FHRC |
| *Flavobacterium columnare* | No | (Boutin et al., 2012) |
| *Klebsiella aerogenes* | No | (Benghezal et al., 2006) |
| *Pseudomonas* sp. 11A isolate | No | (Gauthier et al., 2017a) |
| *Pseudomonas* sp. 11B isolate | No | (Boutin et al., 2012) |
| *Pseudomonas* sp. 13 isolate | No | (Boutin et al., 2012) |
| *Pseudomonas putida* | No | (Berthiaume et al., 2014) |
| *Ralstonia* sp. | No | (Berthiaume et al., 2014) |
| *Vibrio cholerae* 4996 HER1052 | No | FHRC |
| *Vibrio cholerae* 493 HER1354 | No | FHRC |
| *Vibrio natriegens* HER1138 | No | FHRC |
| *Vibrio parahaemolyticus* VP5 HER1169 | No | FHRC |
| *Vibrio parahaemolyticus* HER1193 | No | FHRC |
| *Vibrio vulnificus* HER1289 | No | FHRC |

*FHRC: The Félix d'Hérelle Reference Center for Bacterial Viruses ([www.phage.ulaval.ca](http://www.phage.ulaval.ca)).

**Table S2. Primers used in this study.**

| Primer name | Sequence (5’ to 3’) |
| --- | --- |
| MQM1-F1-1 | CTCCACGTAGGAGCCTAGATAA |
| MQM1-R1-1 | CTGAGAGACGTTGACGAGAATG |
| MQM1-F1-2 | CTTCTCCACCATAGACCAACTG |
| MQM1-R1-2 | CGCAAGGAACCTACTCTGTAAA |
| MQM1-F2-1 | AGGACTATCGGGTGGAAGAT |
| MQM1-R2-1 | GACGACGACGAGGAATTCTAAA |
| MQM1-F2-2 | ACGCCAACGTAGCCATATTC |
| MQM1-R2-2 | CCGTAAGCTGTGCTCTGTAAG |
| MQM1-FA-1 | CCCTCTCTTCTCCCGTAGTAAT |
| MQM1-RA-1 | GAACCCATTAGGGACCCATTAG |
| MQM1-FA-2 | TAACGGTAAGTCCCTCTCTTCT |
| MQM1-RA-2 | GTCCCTTACCCGAACCAAAT |
| MQM1-FB1-1 | CACCCAGTAGTTCGATGAAGTC |
| MQM1-RB1-1 | GTAAGAAGAACCGCCGTATGT |
| MQM1-FB1-2 | GGATTGTTCTTTGATGCCCTTG |
| MQM1-RB1-2 | TCACCGAGTGTGTTGTGATAAT |
| MQM1-FB2-1 | CCGCTCTAACTGTCAACTAAGG |
| MQM1-RB2-1 | TGTATGGAAGGCGTGATTTGTA |
| MQM1-FB2-2 | GCCCTTTGGATGGCTTAAATC |
| MQM1-RB2-2 | GATACACACGGGAATCACACTA |

**Table S3. Predicted functions of the ORF associated with the genes of phage MQM1.**

| **ORF** | **Strand** | **Genomic coordinates** | **Predicted protein** | | | | **Start codon** | **Best match on**  **Blastp** | | | | | **Additional evidence or notes^a^** |
| --- | --- | --- | --- | --- | --- | --- | --- | --- | --- | --- | --- | --- | --- |
|  |  |  | **Size (bp)** | **Size (aa)** | **MW**  **(kDa)** | **GC (%)** |  | **Annotation (predicted function)** | **Identity (%)** | **Coverage (%)** | **E-value** | **Blastp hit** |  |
| 1 | + | 228..602 | 375 | 124 | 13.6 | 54.7 | ATG | Terminase small subunit [uncultured Mediterranean phage uvMED] | 32.8 | 93 | 2e-05 | [BAR34470.1](https://www.ncbi.nlm.nih.gov/protein/BAR34470.1?report=genbank&log$=prottop&blast_rank=3&RID=ZW9K6VXB016) | Same on HHpred ([6W7T_B](https://www.rcsb.org/pdb/explore.do?structureId=6W7T)). |
| 2 | + | 700..774 | 75 | - | 26.7 | 52.0 | - | tRNA-Gln | - | - | - | - | tRNA Scan.SE, Aragorn. |
| 3 | + | 787..863 | 77 | - | 22.3 | 54.5 | - | tRNA-Asn | - | - | - | - | tRNA Scan.SE, Aragorn. |
| 4 | + | 1,088..1,159 | 72 | - | 26.8 | 65.3 | - | tRNA-Met | - | - | - | - | tRNA Scan.SE, Aragorn. |
| 5 | + | 1,250..1,324 | 75 | - | 28.4 | 49.3 | - | tRNA-Pro | - | - | - | - | tRNA Scan.SE, Aragorn. |
| 6 | + | 1,578..1,667 | 90 | - | 30.4 | 55.6 | - | tRNA-Ser | - | - | - | - | tRNA Scan.SE, Aragorn. |
| 7 | + | 1,675..1,749 | 75 | - | 24.1 | 50.7 | - | tRNA-Thr | - | - | - | - | tRNA Scan.SE, Aragorn. |
| 8 | + | 1,753..1,829 | 77 | - | 27.9 | 50.6 | - | tRNA-Arg | - | - | - | - | tRNA Scan.SE, Aragorn. |
| 9 | + | 1,839..1,927 | 89 | - | 31.6 | 55.1 | - | tRNA-Tyr | - | - | - | - | tRNA Scan.SE, Aragorn. |
| 10 | + | 2,382..2,798 | 138 | 417 | 15.1 | 45.1 | GTG | Hypothetical protein [*Vibrio* phage CHOED] | 40.3 | 83 | 2e-24 | [YP_009021753.1](https://www.ncbi.nlm.nih.gov/protein/YP_009021753.1?report=genbank&log$=prottop&blast_rank=1&RID=ZFD7HVNK013) | Spanin2_3 super family ([cl41584](https://www.ncbi.nlm.nih.gov/Structure/cdd/cddsrv.cgi?ascbin=8&maxaln=10&seltype=2&uid=cl41584)) |
| 11 | + | 2,808..4,730 | 1923 | 640 | 73.1 | 50.4 | ATG | Terminase large subunit [*Vibrio* phage CHOED] | 57.8 | 99 | 0.0 | [YP_009021754.1](https://www.ncbi.nlm.nih.gov/protein/YP_009021754.1?report=genbank&log$=prottop&blast_rank=1&RID=ZFDHNDWB016) | Same on HHpred ([5OE8_C](https://www.rcsb.org/pdb/explore.do?structureId=5OE8)).  Domain hit: Terminase_6 super family ([cl40838](https://www.ncbi.nlm.nih.gov/Structure/cdd/cddsrv.cgi?ascbin=8&maxaln=10&seltype=2&uid=cl40838)) |
| 12 | + | 4,739..6,865 | 2127 | 708 | 77.3 | 51.5 | ATG | Portal protein [*Vibrio* phage CHOED] | 62.5 | 99 | 0.0 | [YP_009021755.1](https://www.ncbi.nlm.nih.gov/protein/YP_009021755.1?report=genbank&log$=prottop&blast_rank=1&RID=ZFE5SDDE013) | Same on HHpred ([7SZ6_g](https://www.rcsb.org/pdb/explore.do?structureId=7SZ6)). |
| 13 | + | 6,865..7,077 | 213 | 70 | 78.6 | 51.6 | ATG | Hypothetical protein [*Vibrio* phage CHOED] | 37.3 | 95 | 5e-05 | [YP_009021756.1](https://www.ncbi.nlm.nih.gov/protein/YP_009021756.1?report=genbank&log$=prottop&blast_rank=1&RID=ZFEAAC3J016) | - |
| 14 | + | 7,181..7,942 | 762 | 253 | 28.2 | 51.7 | ATG | Hypothetical protein [*Vibrio* phage CHOED] | 45.6 | 98 | 5e-52 | [YP_009021757.1](https://www.ncbi.nlm.nih.gov/protein/YP_009021757.1?report=genbank&log$=prottop&blast_rank=1&RID=ZFEE4HZH013) | - |
| 15 | + | 7,960..9,093 | 1134 | 377 | 40.4 | 50.9 | ATG | Major capsid protein [*Vibrio* phage CHOED] | 71.9 | 100 | 0.0 | [YP_009021758.1](https://www.ncbi.nlm.nih.gov/protein/YP_009021758.1?report=genbank&log$=prottop&blast_rank=1&RID=ZFETTBE9016) | Same on HHpred ([3J7W_G](http://www.rcsb.org/pdb/explore/explore.do?structureId=3J7W)). |
| 16 | + | 9,171..10,733 | 1563 | 520 | 56.2 | 48.0 | ATG | Hypothetical protein [*Vibrio* phage CHOED] | 42.7 | 100 | 1e-132 | [YP_009021759.1](https://www.ncbi.nlm.nih.gov/protein/YP_009021759.1?report=genbank&log$=prottop&blast_rank=1&RID=ZFEZ91ZT013) | - |
| 17 | + | 10,730..11,902 | 1173 | 390 | 41.6 | 49.2 | ATG | Hypothetical protein [*Vibrio* phage CHOED] | 61.0 | 15 | 4e-13 | [YP_009021760.1](https://www.ncbi.nlm.nih.gov/protein/YP_009021760.1?report=genbank&log$=prottop&blast_rank=1&RID=ZFF33JN2016) | - |
| 18 | + | 11,981..12,691 | 711 | 236 | 27.4 | 48.2 | ATG | Tail protein [*Vibrio* phage CHOED] | 67.8 | 100 | 6e-118 | [YP_009021671.1](https://www.ncbi.nlm.nih.gov/protein/YP_009021671.1?report=genbank&log$=prottop&blast_rank=1&RID=ZFF7278G016) | HHpred hit: [7Z4A_N](http://www.rcsb.org/pdb/explore/explore.do?structureId=7Z4A)  Bacteriophage SU10 tail and bottom part of the capsid (C1) (prob=99.9, e-value= 1.7e-21). |
| 19 | + | 12,693..14,255 | 1563 | 520 | 56.8 | 48.0 | ATG | Amidase [*Vibrio* phage CHOED] | 57.5 | 99 | 0.0 | [YP_009021672.1](https://www.ncbi.nlm.nih.gov/protein/YP_009021672.1?report=genbank&log$=prottop&blast_rank=1&RID=ZFFDKAHE013) | HHpred hit: [7EEQ_5](https://www.rcsb.org/pdb/explore.do?structureId=7EEQ)  (Cyanophage Pam1 tail machine (prob.=100, e-value= 1.9e-39). |
| 20 | + | 14,252..14,467 | 216 | 71 | 76.8 | 47.7 | ATG | Hypothetical protein [*Vibrio* phage CHOED] | 38.8 | 94 | 6e-05 | [YP_009021673.1](https://www.ncbi.nlm.nih.gov/protein/YP_009021673.1?report=genbank&log$=prottop&blast_rank=1&RID=ZFFM19M4016) | - |
| 21 | + | 14,467..14,790 | 324 | 107 | 11.7 | 45.1 | ATG | Hypothetical protein [*Vibrio* phage CHOED] | 45.3 | 98 | 1e-22 | [YP_009021674.1](https://www.ncbi.nlm.nih.gov/protein/YP_009021674.1?report=genbank&log$=prottop&blast_rank=1&RID=ZFG01J72013) | HHpred hit: [3UH8_A](https://www.rcsb.org/pdb/explore.do?structureId=3UH8).N-terminal domain of phage TP901-1 ORF48 (prob.=98.3, e-value= 3.2e-4). |
| 22 | + | 14,790..16,025 | 1236 | 411 | 43.2 | 50.8 | ATG | Tail fiber protein [*Vibrio* phage CHOED] | 52.0 | 72 | 4e-43 | [YP_009021675.1](https://www.ncbi.nlm.nih.gov/protein/YP_009021675.1?report=genbank&log$=prottop&blast_rank=1&RID=ZFG5820W013) | - |
| 23 | + | 16,035..16,592 | 558 | 185 | 19.7 | 49.6 | ATG | Hypothetical protein [*Methylacidiphilales* bacterium] | 70.2 | 25 | 3e-11 | [MCE0484361.1](https://www.ncbi.nlm.nih.gov/protein/MCE0484361.1?report=genbank&log$=prottop&blast_rank=1&RID=ZFHA90KE016) | HHpred hit: [PF18454.4](http://pfam-legacy.xfam.org/family/PF18454.4)  Major tropism determinant N-terminal domain (prob.=99.1, e-value=4.4e-10). Domain hit: Mtd_N superfamily ([pfam18454](https://www.ncbi.nlm.nih.gov/Structure/cdd/cddsrv.cgi?ascbin=8&maxaln=10&seltype=2&uid=pfam18454)). |
| 24 | + | 16,605..17,306 | 702 | 233 | 24.0 | 52.1 | ATG | Hypothetical protein | - | - | - | - | - |
| 25 | + | 17,318..18,754 | 1437 | 478 | 47.2 | 55.0 | ATG | Hypothetical protein | - | - | - | - | - |
| 26 | + | 18,823..20,355 | 1533 | 510 | 53.6 | 52.6 | ATG | Hypothetical protein [*Vibrio* phage CHOED] | 60.0 | 75 | 6e-139 | [YP_009021679.1](https://www.ncbi.nlm.nih.gov/protein/YP_009021679.1?report=genbank&log$=prottop&blast_rank=1&RID=ZFJ14KPW01R) | - |
| 27 | + | 20,408..20,842 | 435 | 144 | 15.2 | 52.0 | ATG | Hypothetical protein [*Vibrio* phage CHOED] | 48.5 | 93 | 4e-31 | [YP_009021680.1](https://www.ncbi.nlm.nih.gov/protein/YP_009021680.1?report=genbank&log$=prottop&blast_rank=1&RID=ZFJ64AXV013) | - |
| 28 | + | 20,842..21,402 | 561 | 186 | 20.3 | 53.5 | ATG | Hypothetical protein [*Vibrio* phage CHOED] | 46.3 | 99 | 3e-51 | [YP_009021681.1](https://www.ncbi.nlm.nih.gov/protein/YP_009021681.1?report=genbank&log$=prottop&blast_rank=1&RID=ZHNFRGTK013) | - |
| 29 | + | 21,404..23,251 | 1848 | 615 | 67.5 | 52.2 | ATG | Hypothetical protein [*Vibrio* phage CHOED] | 29.9 | 21 | 4e-06 | [YP_009021682.1](https://www.ncbi.nlm.nih.gov/protein/YP_009021682.1?report=genbank&log$=prottop&blast_rank=1&RID=ZJ7NHZ0701N) | - |
| 30 | + | 23,262..25,718 | 2457 | 818 | 88.0 | 51.9 | ATG | Hypothetical protein | 21.4 | 75.7 | 9.71e-31 | YP_009021683.1 | - |
| 31 | + | 25,782..26,039 | 258 | 85 | 94.7 | 45.7 | ATG | Hypothetical protein | - | - | - | - | HHpred hit: [PF10779.12](http://pfam-legacy.xfam.org/family/PF10779.12)  Haemolysin XhlA(probability=98.5, e-value= 1.5e-5). |
| 32 | - | 26,232..26,693 | 462 | 153 | 17.6 | 47.8 | ATG | Hypothetical protein | - | - | - | - | - |
| 33 | - | 26,690..27,286 | 597 | 198 | 21.8 | 53.6 | ATG | Hypothetical protein | - | - | - | - | - |
| 34 | - | 27,393..27,638 | 246 | 81 | 93.4 | 46.7 | ATG | Hypothetical protein [*Vibrio* phage CHOED] | 45.3 | 74 | 4e-07 | [YP_009021685.1](https://www.ncbi.nlm.nih.gov/protein/YP_009021685.1?report=genbank&log$=prottop&blast_rank=1&RID=ZSFB084G016) | - |
| 35 | - | 27,648..27,890 | 243 | 80 | 89.3 | 54.7 | ATG | Hypothetical protein | - | - | - | - | - |
| 36 | - | 28,008..28,163 | 156 | 51 | 62.2 | 50.0 | ATG | Hypothetical protein | - | - | - | - | - |
| 37 | - | 28,225..29,292 | 1068 | 355 | 40.3 | 51.4 | ATG | ATP-dependent DNA ligase [*Vibrio* phage CHOED] | 45.1 | 96 | 1e-86 | [YP_009021689.1](https://www.ncbi.nlm.nih.gov/protein/YP_009021689.1?report=genbank&log$=prottop&blast_rank=1&RID=ZSGKEYCW01N) | Same on HHpred ([6IML_A](https://www.rcsb.org/pdb/explore.do?structureId=6IML)).  Domain hit: 30 super family ([cl33690](https://www.ncbi.nlm.nih.gov/Structure/cdd/cddsrv.cgi?ascbin=8&maxaln=10&seltype=2&uid=cl33690)) |
| 38 | - | 29,332..29,883 | 552 | 183 | 20.8 | 51.3 | ATG | Hypothetical protein [*Vibrio* phage CHOED] | 36 | 81 | 7e-20 | [YP_009021691.1](https://www.ncbi.nlm.nih.gov/protein/YP_009021691.1?report=genbank&log$=prottop&blast_rank=1&RID=ZSGYT74N016) | HHpred hit: [5I8U_C](https://www.rcsb.org/pdb/explore.do?structureId=5I8U)  ADP-ribose pyrophosphatase  (probability=99.8, e-value= 4.5e-17). Domain hit: ADPRase_NUDT5 ([cd03424](https://www.ncbi.nlm.nih.gov/Structure/cdd/cddsrv.cgi?ascbin=8&maxaln=10&seltype=2&uid=cd03424)) |
| 39 | - | 29,861..30,439 | 579 | 192 | 21.7 | 48.7 | ATG | Thymidine kinase [*Vibrio* phage CHOED] | 58.4 | 94 | 7e-71 | [YP_009021692.1](https://www.ncbi.nlm.nih.gov/protein/YP_009021692.1?report=genbank&log$=prottop&blast_rank=1&RID=ZSHC2GGS013) | Same on HHpred ([2B8T_A](https://www.rcsb.org/pdb/explore.do?structureId=2B8T)).  Domain hit: TK super family ([cl23762](https://www.ncbi.nlm.nih.gov/Structure/cdd/cddsrv.cgi?ascbin=8&maxaln=10&seltype=2&uid=cl23762)) |
| 40 | - | 30,429..30,827 | 399 | 132 | 15.0 | 43.9 | TTG | HNH endonuclease [*Pseudomonas oleovorans*] | 43.8 | 87 | 6e-23 | [WP_150606293.1](https://www.ncbi.nlm.nih.gov/protein/WP_150606293.1?report=genbank&log$=prottop&blast_rank=3&RID=0F5B3M3X013) | HHpred hit: [1A73_B](https://www.rcsb.org/pdb/explore.do?structureId=1A73)  Intron-encoded endonuclease I-PPOI complexed with DNA.  (probability=99.6, e-value= 8.3e-15). Domain hits: HNHc super family ([cl00083](https://www.ncbi.nlm.nih.gov/Structure/cdd/cddsrv.cgi?ascbin=8&maxaln=10&seltype=2&uid=cl00083))  HTH_XRE ([cd00093](https://www.ncbi.nlm.nih.gov/Structure/cdd/cddsrv.cgi?ascbin=8&maxaln=10&seltype=2&uid=cd00093)) |
| 41 | - | 30,944..31,114 | 171 | 56 | 64.7 | 45.0 | ATG | Hypothetical protein | - | - | - | - | - |
| 42 | - | 31,176..31,796 | 621 | 206 | 23.8 | 50.6 | ATG | Deoxynucleoside kinase [*Sansalva-dorimonas* sp. 2012CJ34-2] | 65.6 | 89 | 3e-87 | [WP_249697632.1](https://www.ncbi.nlm.nih.gov/protein/WP_249697632.1?report=genbank&log$=prottop&blast_rank=1&RID=ZTWP32TP016) | Same on HHpred ([2JAQ_A](https://www.rcsb.org/pdb/explore.do?structureId=2JAQ)).  Domain hit: NK super family ([cl17190](https://www.ncbi.nlm.nih.gov/Structure/cdd/cddsrv.cgi?ascbin=8&maxaln=10&seltype=2&uid=cl17190)). |
| 43 | - | 31,798..31,986 | 189 | 62 | 71.8 | 48.1 | ATG | Hypothetical protein | - | - | - | - | - |
| 44 | - | 31,983..32,198 | 216 | 71 | 74.8 | 50.5 | ATG | Hypothetical protein | - | - | - | - | - |
| 45 | - | 32,195..32,929 | 735 | 244 | 27.6 | 51.6 | ATG | Deoxynucleoside-5-monophosphate kinase [*Vibrio* phage CHOED] | 46.9 | 97 | 3e-59 | [YP_009021697.1](https://www.ncbi.nlm.nih.gov/protein/YP_009021697.1?report=genbank&log$=prottop&blast_rank=1&RID=ZTWZGKDY016) | Same on HHpred ([1DEK_A](https://www.rcsb.org/pdb/explore.do?structureId=1DEK)).  Domain hit: NK super family ([cl17190](https://www.ncbi.nlm.nih.gov/Structure/cdd/cddsrv.cgi?ascbin=8&maxaln=10&seltype=2&uid=cl17190)). |
| 46 | - | 32,931..33,152 | 222 | 73 | 81.9 | 46.4 | ATG | Hypothetical protein | - | - | - | - | - |
| 47 | - | 33,172..33,684 | 513 | 170 | 19.5 | 48.5 | ATG | Hypothetical protein [*Vibrio* phage CHOED] | 58.1 | 91 | 3e-55 | [YP_009021699.1](https://www.ncbi.nlm.nih.gov/protein/YP_009021699.1?report=genbank&log$=prottop&blast_rank=1&RID=ZUP31PJW01N) | HHpred hit: [2H8E_A](https://www.rcsb.org/pdb/explore.do?structureId=2H8E)  Crossover junction endodeoxribonuclease RusA (probability=99.8, e-value= 2.9e-17). Domain hit: RusA super family ([cl01885](https://www.ncbi.nlm.nih.gov/Structure/cdd/cddsrv.cgi?ascbin=8&maxaln=10&seltype=2&uid=cl01885)) |
| 48 | - | 33,782..34,846 | 1065 | 354 | 40.5 | 47.4 | ATG | 5' -3' exonuclease [*Vibrio* phage CHOED] | 68.2 | 96 | 1e-173 | [YP_009021700.1](https://www.ncbi.nlm.nih.gov/protein/YP_009021700.1?report=genbank&log$=prottop&blast_rank=1&RID=ZUPBVPU8013) | Same on HHpred ([6C33_A](https://www.rcsb.org/pdb/explore.do?structureId=6C33)).  Domain hit: PHA00439 super family ([cl33674](https://www.ncbi.nlm.nih.gov/Structure/cdd/cddsrv.cgi?ascbin=8&maxaln=10&seltype=2&uid=cl33674)) |
| 49 | - | 34,843..35,298 | 456 | 151 | 16.8 | 51.5 | ATG | DNA endonuclease [*Enterococcus* phage EF36P1] | 37.9 | 100 | 1e-21 | [WAX14850.1](https://www.ncbi.nlm.nih.gov/protein/WAX14850.1?report=genbank&log$=prottop&blast_rank=5&RID=ZUPKATDK013) | HHpred hit: [1U3E_M](https://www.rcsb.org/pdb/explore.do?structureId=1U3E)  HNH homing endonuclease (probability=100, e-value= 2.9e-30). Domain hit: HNHc superfamily ([cl00083](https://www.ncbi.nlm.nih.gov/Structure/cdd/cddsrv.cgi?ascbin=8&maxaln=10&seltype=2&uid=cl00083)). |
| 50 | - | 35,335..35,523 | 189 | 62 | 71.3 | 55.0 | ATG | Hypothetical protein | - | - | - | - | - |
| 51 | - | 35,601..35,825 | 225 | 74 | 85.2 | 46.7 | ATG | Hypothetical protein *Pseudoalteromonas* | 67.6 | 100 | 2e-28 | [WP_211012411.1](https://www.ncbi.nlm.nih.gov/protein/WP_211012411.1?report=genbank&log$=prottop&blast_rank=1&RID=ZURB01WF016) | - |
| 52 | - | 35.827..36.432 | 606 | 201 | 22.8 | 49.7 | ATG | Hypothetical protein [*Pseudoalteromonas* phage J2-1] | 29.7 | 90 | 3e-07 | [ATN93488.1](https://www.ncbi.nlm.nih.gov/protein/ATN93488.1?report=genbank&log$=prottop&blast_rank=1&RID=ZURHPXR4016) | HHpred hit: [1MIW_B](https://www.rcsb.org/pdb/explore.do?structureId=1MIW)  tRNA CCA-adding enzyme (probability=99.9, e-value= 1.1e-21). Domain hit: PolyA_pol super family ([cl21612](https://www.ncbi.nlm.nih.gov/Structure/cdd/cddsrv.cgi?ascbin=8&maxaln=10&seltype=2&uid=cl21612)) |
| 53 | - | 36,429..36,572 | 144 | 47 | 52.8 | 45.8 | GTG | Hypothetical protein | - | - | - | - | - |
| 54 | - | 36,563..36,754 | 192 | 63 | 68.6 | 49.0 | ATG | Hypothetical protein [*Pseudomonas chlororaphis*] | 50 | 69 | 2e-04 | [WP_039967909.1](https://www.ncbi.nlm.nih.gov/protein/WP_039967909.1?report=genbank&log$=prottop&blast_rank=1&RID=ZURVKMW6013) | - |
| 55 | - | 36,839..40,009 | 2238 | 762 | 83.53 | 52.7 | ATG | DNA polymerase I [*Vibrio* phage CHOED] | 60 | 97 | 0 | [YP_009021707.1](https://www.ncbi.nlm.nih.gov/protein/YP_009021707.1?report=genbank&log$=prottop&blast_rank=1&RID=0HUBBWA1016) | Same on HHpred ([1X9M_A](https://www.rcsb.org/pdb/explore.do?structureId=1X9M)). |
| 56 | - | 40,094..41,833 | 1740 | 579 | 64.4 | 50.3 | ATG | DNA primase/helicase [*Vibrio* phage CHOED] | 74.5 | 99 | 0 | [YP_009021708.1](https://www.ncbi.nlm.nih.gov/protein/YP_009021708.1?report=genbank&log$=prottop&blast_rank=1&RID=ZV3DYYRU016) | Same on HHpred ([6N7I_C](https://www.rcsb.org/pdb/explore.do?structureId=6N7I)).  Domain hits: RecA-like_Gp4D_helicase ([cd19483](https://www.ncbi.nlm.nih.gov/Structure/cdd/cddsrv.cgi?ascbin=8&maxaln=10&seltype=2&uid=cd19483))  TOPRIM super family ([cl00718](https://www.ncbi.nlm.nih.gov/Structure/cdd/cddsrv.cgi?ascbin=8&maxaln=10&seltype=2&uid=cl00718)) |
| 57 | - | 41,843..42,076 | 234 | 77 | 87.6 | 48.3 | ATG | Hypothetical protein [*Vibrio* phage CHOED] | 47.1 | 88 | 4e-11 | [YP_009021709.1](https://www.ncbi.nlm.nih.gov/protein/YP_009021709.1?report=genbank&log$=prottop&blast_rank=1&RID=ZV3J6M4U013) | - |
| 58 | - | 42,057..42,293 | 237 | 78 | 84.0 | 48.5 | ATG | Hypothetical protein | - | - | - | - | - |
| 59 | - | 42,392..42,907 | 516 | 171 | 18.9 | 54.1 | ATG | Hypothetical protein | - | - | - | - | - |
| 60 | - | 42,927..44,072 | 1146 | 381 | 43.5 | 51.2 | ATG | RNA ligase 2 [*Vibrio* phage CHOED] | 45 | 92 | 7e-93 | [YP_009021711.1](https://www.ncbi.nlm.nih.gov/protein/YP_009021711.1?report=genbank&log$=prottop&blast_rank=1&RID=ZV3V3PUR016) | Same on HHpred ([2HVQ_A](https://www.rcsb.org/pdb/explore.do?structureId=2HVQ)).  Domain hit: T4_Rnl2_C super family ([cl40261](https://www.ncbi.nlm.nih.gov/Structure/cdd/cddsrv.cgi?ascbin=8&maxaln=10&seltype=2&uid=cl40261)) |
| 61 | - | 44,077..44,298 | 222 | 73 | 79.1 | 53.2 | ATG | Hypothetical protein [*Morganella morganii*] | 44.4 | 98 | 3e-06 | [WP_214180943.1](https://www.ncbi.nlm.nih.gov/protein/WP_214180943.1?report=genbank&log$=prottop&blast_rank=1&RID=ZV3ZCX4D013) | - |
| 62 | - | 44,314..44,559 | 246 | 81 | 88.9 | 52.8 | ATG | Hypothetical protein [*Vibrio* phage CHOED] | 48.7 | 88 | 1e-12 | [YP_009021714.1](https://www.ncbi.nlm.nih.gov/protein/YP_009021714.1?report=genbank&log$=prottop&blast_rank=1&RID=ZV4438ZM013) | - |
| 63 | - | 44,730..45,203 | 474 | 157 | 16.7 | 51.1 | ATG | Hypothetical protein | - | - | - | - | - |
| 64 | - | 45,448..45,786 | 339 | 112 | 12.5 | 53.1 | ATG | Hypothetical protein | - | - | - | - | - |
| 65 | - | 45,842..47,083 | 1242 | 413 | 45.0 | 54.4 | ATG | SPFH domain-containing protein [unclassified *Salinivibrio*] | 44.8 | 97 | 4e-104 | [WP_158010255.1](https://www.ncbi.nlm.nih.gov/protein/WP_158010255.1?report=genbank&log$=prottop&blast_rank=1&RID=ZV571RCW016) | Same on HHpred ([7VHP_S](https://www.rcsb.org/pdb/explore.do?structureId=7VHP)).  Domain hits: ATP-synt_Fo_b super family ([cl38961](https://www.ncbi.nlm.nih.gov/Structure/cdd/cddsrv.cgi?ascbin=8&maxaln=10&seltype=2&uid=cl38961))  TolA super family ([cl34546](https://www.ncbi.nlm.nih.gov/Structure/cdd/cddsrv.cgi?ascbin=8&maxaln=10&seltype=2&uid=cl34546)) |
| 66 | - | 47,143..47,475 | 333 | 110 | 12.5 | 52.6 | ATG | Hypothetical protein | - | - | - | - | - |
| 67 | - | 47,485..47,862 | 378 | 125 | 13.8 | 51.9 | ATG | Hypothetical protein | - | - | - | - | - |
| 68 | - | 47,883..48,320 | 438 | 145 | 15.4 | 52.3 | ATG | Hypothetical protein | - | - | - | - | - |
| 69 | - | 48,351..48,548 | 198 | 68 | 70.7 | 50.0 | ATG | Hypothetical protein [*Aeromonas* phage 4_4572] | 67.2 | 89.2 | 7e-17 | [YP_009847147.1](https://www.ncbi.nlm.nih.gov/protein/YP_009847147.1?report=genbank&log$=prottop&blast_rank=1&RID=ZV5YY6EE016) | - |
| 70 | - | 48,584..48,943 | 360 | 119 | 13.2 | 49.2 | ATG | Hypothetical protein | - | - | - | - | - |
| 71 | - | 49,023..49,223 | 201 | 66 | 75.9 | 48.3 | TTG | Hypothetical protein | - | - | - | - | - |
| 72 | - | 49,225..49,731 | 507 | 168 | 18.6 | 51.9 | ATG | Hypothetical protein [*Vibrio* phage CHOED] | 76.1 | 92 | 4e-85 | [YP_009021730.1](https://www.ncbi.nlm.nih.gov/protein/YP_009021730.1?report=genbank&log$=prottop&blast_rank=1&RID=ZV6AWVUN013) | HHpred hit: [5ODJ_A](https://www.rcsb.org/pdb/explore.do?structureId=5ODJ)  Single-stranded DNA-binding protein (prob.=99.9, e-value= 7.2e-23). |
| 73 | - | 49,885..50,376 | 492 | 163 | 17.4 | 56.9 | GTG | Phosphoribosylformylglycinamidine synthase I [*Planctomycetaceae*] | 34.3 | 61 | 0.036 | [MCA8985337.1](https://www.ncbi.nlm.nih.gov/protein/MCA8985337.1?report=genbank&log$=prottop&blast_rank=1&RID=ZV6HC8JZ013) | - |
| 74 | - | 50,464..51,027 | 564 | 187 | 20.1 | 54.8 | ATG | Hypothetical protein | - | - | - | - | - |
| 75 | - | 51,241..51,462 | 222 | 73 | 84.6 | 50.5 | ATG | Hypothetical protein [*Escherichia coli*] | 50 | 83 | 2e-12 | [WP_236452099.1](https://www.ncbi.nlm.nih.gov/protein/WP_236452099.1?report=genbank&log$=prottop&blast_rank=1&RID=ZV714E6901N) | - |
| 76 | - | 51,523..51,759 | 237 | 78 | 87.1 | 50.6 | ATG | Hypothetical protein | - | - | - | - | - |
| 77 | - | 51,756..52,001 | 248 | 81 | 92.2 | 47.6 | GTG | Hypothetical protein | - | - | - | - | - |
| 78 | - | 51,995..52,567 | 573 | 190 | 21.0 | 51.7 | ATG | Lysozyme [*Vibrio cholerae*] | 39.1 | 95 | 8e-33 | [WP_199352586.1](https://www.ncbi.nlm.nih.gov/protein/WP_199352586.1?report=genbank&log$=prottop&blast_rank=1&RID=ZV7ND96M016) | Same on HHpred ([6ET6_A](https://www.rcsb.org/pdb/explore.do?structureId=6ET6)).  Domain hit: Lyz-like super family ([cl00222](https://www.ncbi.nlm.nih.gov/Structure/cdd/cddsrv.cgi?ascbin=8&maxaln=10&seltype=2&uid=cl00222)) |
| 79 | - | 52,809..53,408 | 600 | 199 | 21.9 | 50.0 | ATG | Hypothetical protein [*Aeromonas* phage LAh_6] | 40.9 | 97 | 5e-38 | [YP_009847315.1](https://www.ncbi.nlm.nih.gov/protein/YP_009847315.1?report=genbank&log$=prottop&blast_rank=1&RID=ZV8EPFPY016) | - |
| 80 | - | 53,411..53,695 | 285 | 94 | 10.4 | 50.5 | ATG | Thioredoxin TrxC [*Magnetofaba australis*] | 35 | 85 | 2e-09 | [WP_085440098.1](https://www.ncbi.nlm.nih.gov/protein/WP_085440098.1?report=genbank&log$=prottop&blast_rank=1&RID=ZV8UYU0P01N) | Same on HHpred ([3GNJ_C](https://www.rcsb.org/pdb/explore.do?structureId=3GNJ)).  Domain hit: TRX_family ([cd02947](https://www.ncbi.nlm.nih.gov/Structure/cdd/cddsrv.cgi?ascbin=8&maxaln=10&seltype=2&uid=cd02947)) |
| 81 | - | 53,679..53,864 | 186 | 61 | 66.8 | 50.5 | ATG | Hypothetical protein [*Prevotella* sp.] | 43.8 | 72 | 0.011 | [MBP5425090.1](https://www.ncbi.nlm.nih.gov/protein/MBP5425090.1?report=genbank&log$=prottop&blast_rank=1&RID=ZV937455016) | - |
| 82 | - | 53,866..54,126 | 261 | 86 | 95.3 | 47.1 | TTG | Hypothetical protein | - | - | - | - | - |
| 83 | - | 54,176..54,373 | 198 | 65 | 72.2 | 47.5 | ATG | Hypothetical protein | - | - | - | - | - |
| 84 | - | 54,564..55,043 | 480 | 159 | 18.2 | 51.9 | ATG | Hypothetical protein | 55.9 | 100 | 2.62e-55 | - | HHpred hit: [PF03013.17](http://pfam-legacy.xfam.org/family/PF03013.17)  Pyrimidine dimer DNA glycosylase (probability=99.8, e-value= 1.5e-18). |
| 85 | - | 55,024..55,245 | 222 | 73 | 87.0 | 41.9 | ATG | Hypothetical protein | - | - | - | - | - |
| 86 | - | 55,253..56,350 | 1098 | 365 | 40.8 | 51.6 | ATG | RNA polymerase [*Vibrio* phage CHOED] | 57 | 100 | 2e-133 | [YP_009021742.2](https://www.ncbi.nlm.nih.gov/protein/YP_009021742.2?report=genbank&log$=prottop&blast_rank=1&RID=ZW64SND0016) | Same on HHpred ([1MSW_D](https://www.rcsb.org/pdb/explore.do?structureId=1MSW)).  PHA00452 super family ([cl42978](https://www.ncbi.nlm.nih.gov/Structure/cdd/cddsrv.cgi?ascbin=8&maxaln=10&seltype=2&uid=cl42978)). Domain hit: PHA00452 super family ([cl42978](https://www.ncbi.nlm.nih.gov/Structure/cdd/cddsrv.cgi?ascbin=8&maxaln=10&seltype=2&uid=cl42978)) |
| 87 | - | 56,371..58,185 | 1815 | 604 | 69.0 | 48.5 | ATG | RNA polymerase [*Vibrio* phage CHOED] | 51.4 | 95 | 0 | [YP_009021742.2](https://www.ncbi.nlm.nih.gov/protein/YP_009021742.2?report=genbank&log$=prottop&blast_rank=1&RID=ZW6CFS7U013) | Same on HHpred ([1MSW_D](https://www.rcsb.org/pdb/explore.do?structureId=1MSW)).  Domain hit: PHA00452 super family ([cl42978](https://www.ncbi.nlm.nih.gov/Structure/cdd/cddsrv.cgi?ascbin=8&maxaln=10&seltype=2&uid=cl42978)). |
| 88 | - | 58,542..58,838 | 297 | 98 | 11.1 | 42.4 | ATG | Hypothetical protein | - | - | - | - | - |
| 89 | - | 58,841..58,993 | 153 | 50 | 60.4 | 41.2 | ATG | Hypothetical protein | - | - | - | - | - |
| 90 | - | 58,990..59,181 | 192 | 63 | 73.4 | 38.5 | ATG | Hypothetical protein | - | - | - | - | - |
| 91 | - | 59,230..59,484 | 255 | 84 | 90.5 | 45.5 | ATG | Hypothetical protein | - | - | - | - | - |
| 92 | - | 59,559..60,680 | 1122 | 373 | 41.3 | 52.6 | ATG | Streptomycin biosynthesis regulatory protein [*Vibrio* phage CHOED] | 49.5 | 100 | 9e-116 | [YP_009021749.1](https://www.ncbi.nlm.nih.gov/protein/YP_009021749.1?report=genbank&log$=prottop&blast_rank=1&RID=ZW7BR5V4013) | HHpred hit: [7BNR_A](https://www.rcsb.org/pdb/explore.do?structureId=7BNR)  ParB family protein; DNA-binding protein (probability=99.8, e-value=3.7e-17). |
| 93 | - | 60,811..60,996 | 186 | 61 | 70.5 | 49.5 | ATG | Hypothetical protein | - | - | - | - | - |
| 94 | - | 61,118..61,342 | 225 | 74 | 80.7 | 53.8 | ATG | Hypothetical protein [*Vibrio* phage F23s1] | 59.5 | 56 | 2e-06 | [UCW44097.1](https://www.ncbi.nlm.nih.gov/protein/UCW44097.1?report=genbank&log$=prottop&blast_rank=1&RID=ZW7T8KP6016) | - |
| 95 | - | 62,151..62,291 | 141 | 46 | 50.3 | 50.4 | ATG | Hypothetical protein | - | - | - | - | - |
| 96 | + | 62,929..63,342 | 414 | 138 | 15.8 | 45.9 | ATG | Endonuclease [*Halorubrum* tailed virus 29] | 31 | 98 | 2e-10 | [YP_010358430.1](https://www.ncbi.nlm.nih.gov/protein/YP_010358430.1?report=genbank&log$=prottop&blast_rank=1&RID=ZW93SJ65016) | Same on HHpred ([1U3E_M](http://www.rcsb.org/pdb/explore/explore.do?structureId=1U3E)). |

a: Blastp (<https://blast.ncbi.nlm.nih.gov/Blast.cgi>) and HHpred (https://toolkit.tuebingen.mpg.de/tools/hhpred) were used for protein function prediction (Zimmermann et al., 2018). The conserved domains of each predicted ORF were analysed through <https://www.ncbi.nlm.nih.gov/Structure/cdd/wrpsb.cgi>.

**Table S4. tRNAs predicted in the genome of phage MQM1^a^.**

| **tRNA** | **Coding amino acid** | **Anticodon sequence** | **Location on sequence** | **Strand** | **GC%** |
| --- | --- | --- | --- | --- | --- |
| tRNA-Gln | Glutamine | ttg | 700..774 (75 bp) | + | 52.0 |
| tRNA-Asn | Asparagine | gtt | 787..863 (77 bp) | + | 54.5 |
| tRNA-Met | Methionine | cat | 1088..1159 (72 bp) | + | 65.3 |
| tRNA-Pro | Proline | tgg | 1250..1324 (75 bp) | + | 49.3 |
| tRNA-Ser | Serine | gct | 1578..1667 (90 bp) | + | 55.6 |
| tRNA-Thr | Threonine | tgt | 1675..1749 (75 bp) | + | 50.7 |
| tRNA-Arg | Arginine | tct | 1753..1829 (77 bp) | + | 50.6 |
| tRNA-Tyr | Tyrosine | gta | 1839..1927 (89 bp) | + | 55.1 |

a: The tRNA prediction was done using Aragorn and tRNAscan-SE 2.0 (Laslett and Canback, 2004; Lowe and Eddy, 1996).

References

Attéré, S.A., Gagné-Thivierge, C., Paquet, V.E., Leduc, G.R., Vincent, A.T., Charette, S.J., 2023. *Aeromonas salmonicida* isolates from Canada demonstrate wide distribution and clustering among mesophilic strains. Genome. https://doi.org/10.1139/gen-2022-0086

Benghezal, M., Fauvarque, M.O., Tournebize, R., Froquet, R., Marchetti, A., Bergeret, E., Lardy, B., Klein, G., Sansonetti, P., Charette, S.J., Cosson, P., 2006. Specific host genes required for the killing of *Klebsiella* bacteria by phagocytes. Cell. Microbiol. 8, 139–148. https://doi.org/10.1111/j.1462-5822.2005.00607.x

Berthiaume, C., Gilbert, Y., Fournier-Larente, J., Pluchon, C., Filion, G., Jubinville, E., Sérodes, J.B., Rodriguez, M., Duchaine, C., Charette, S.J., 2014. Identification of dichloroacetic acid degrading *Cupriavidus* bacteria in a drinking water distribution network model. J. Appl. Microbiol. 116, 208–221. https://doi.org/10.1111/jam.12353

Boutin, S., Bernatchez, L., Audet, C., Derôme, N., 2012. Antagonistic effect of indigenous skin bacteria of brook charr (*Salvelinus fontinalis*) against *Flavobacterium columnare* and *F. psychrophilum*. Vet. Microbiol. 155, 355–361. https://doi.org/10.1016/j.vetmic.2011.09.002

Burr, S.E., Frey, J., 2007. Analysis of type III effector genes in typical and atypical *Aeromonas salmonicida*. J. Fish Dis. 30, 711–714. https://doi.org/10.1111/j.1365-2761.2007.00859.x

Burr, S.E., Pugovkin, D., Wahli, T., Segner, H., Frey, J., 2005. Attenuated virulence of an *Aeromonas salmonicida* subsp. *salmonicida* type III secretion mutant in a rainbow trout model. Microbiology 151, 2111–2118. https://doi.org/10.1099/mic.0.27926-0

Gauthier, J., Charette, S.J., Derome, N., 2017a. Draft genome sequence of *Pseudomonas fluorescens* ML11A, an endogenous strain from brook charr with antagonistic properties against *Aeromonas salmonicida* subsp. *salmonicida*. Genome Announc. 5, 9–10. https://doi.org/10.1128/genomeA.01716-16

Gauthier, J., Vincent, A.T., Charette, S.J., Derome, N., 2017b. Strong genomic and phenotypic heterogeneity in the *Aeromonas sobria* species complex. Front. Microbiol. 8, 2434. https://doi.org/10.3389/fmicb.2017.02434

Goldschmidt-Clermont, E., Hochwartner, O., Demarta, A., Caminada, A.P., Frey, J., 2009. Outbreaks of an ulcerative and haemorrhagic disease in Arctic char *Salvelinus alpinus* caused by *Aeromonas salmonicida* subsp. *smithia*. Dis. Aquat. Organ. 86, 81–86. https://doi.org/10.3354/dao02110

Küpfer, M., Kuhnert, P., Korczak, B.M., Peduzzi, R., Demarta, A., 2006. Genetic relationships of *Aeromonas* strains inferred from 16S rRNA, *gyrB* and *rpoB* gene sequences. Int. J. Syst. Evol. Microbiol. 56, 2743–2751. https://doi.org/10.1099/ijs.0.63650-0

Laslett, D., Canback, B., 2004. ARAGORN, a program to detect tRNA genes and tmRNA genes in nucleotide sequences. Nucleic Acids Res. 32, 11–16. https://doi.org/10.1093/nar/gkh152

Lopes, A., Tavares, P., Petit, M.A., Guérois, R., Zinn-Justin, S., 2014. Automated classification of tailed bacteriophages according to their neck organization. BMC Genomics 15, 1027. https://doi.org/10.1186/1471-2164-15-1027

Lowe, T.M., Eddy, S.R., 1996. TRNAscan-SE: A program for improved detection of transfer RNA genes in genomic sequence. Nucleic Acids Res. 25, 955–964. https://doi.org/0.1093/nar/25.5.955

Miñana-Galbis, D., Farfán, M., Fusté, M.C., Lorén, J.G., 2004. *Aeromonas molluscorum* sp. nov., isolated from bivalve molluscs. Int. J. Syst. Evol. Microbiol. 54, 2073–2078. https://doi.org/10.1099/ijs.0.63202-0

Nagar, V., Shashidhar, R., Bandekar, J.R., 2011. Prevalence, characterization, and antimicrobial resistance of *Aeromonas* strains from various retail food products in Mumbai, India. J. Food Sci. 76, 486–492. https://doi.org/10.1111/j.1750-3841.2011.02303.x

Nikapitiya, C., Dananjaya, S.H.S., Chandrarathna, H.P.S.U., Senevirathne, A., De Zoysa, M., Lee, J., 2019. Isolation and characterization of multidrug resistance *Aeromonas salmonicida* subsp. *salmonicida* and its infecting novel phage ASP-1 from Goldfish (*Carassius auratu*s). Indian J. Microbiol. 59, 161–170. https://doi.org/10.1007/s12088-019-00782-5

Pavan, M.E., Abbott, S.L., Zorzópulos, J., Janda, J.M., 2000. *Aeromonas salmonicida* subsp. *pectinolytica* subsp. nov., a new pectinase- positive subspecies isolated from a heavily polluted river. Int. J. Syst. Evol. Microbiol. 50, 1119–1124. https://doi.org/10.1099/00207713-50-3-1119

Rouleau, F.D., Vincent, A.T., Charette, S.J., 2018. Genomic and phenotypic characterization of an atypical *Aeromonas salmonicida* strain isolated from a lumpfish and producing unusual granular structures. J. Fish Dis. 41, 673–681. https://doi.org/10.1111/jfd.12769

Studer, N., Frey, J., Vanden Bergh, P., 2013. Clustering subspecies of *Aeromonas salmonicida* using IS630 typing. BMC Microbiol. 13. https://doi.org/10.1186/1471-2180-13-36

Vincent, A.T., Bernatchez, A., Frey, J., Charette, S.J., 2019. A mesophilic *Aeromonas salmonicida* strain isolated from an unsuspected host, the migratory bird pied avocet. Microorganisms 7, 592. https://doi.org/10.3390/microorganisms7120592

Vincent, A.T., Trudel, M. V., Paquet, V.E., Boyle, B., Tanaka, K.H., Dallaire-Dufresne, S., Daher, R.K., Frenette, M., Derome, N., Charette, S.J., 2014. Detection of variants of the pRAS3, pAB5S9, and pSN254 plasmids in *Aeromonas salmonicida* subsp. *salmonicida*: Multidrug resistance, interspecies exchanges, and plasmid reshaping. Antimicrob. Agents Chemother. 58, 7367–7374. https://doi.org/10.1128/AAC.03730-14

Zimmermann, L., Stephens, A., Nam, S.Z., Rau, D., Kübler, J., Lozajic, M., Gabler, F., Söding, J., Lupas, A.N., Alva, V., 2018. A completely reimplemented MPI bioinformatics toolkit with a new HHpred server at its core. J. Mol. Biol. 430, 2237–2243. https://doi.org/10.1016/j.jmb.2017.12.007
